# *In situ* characterizing membrane lipid phenotype of breast cancer cells using mass spectrometry profiling

**DOI:** 10.1038/srep11298

**Published:** 2015-06-10

**Authors:** Manwen He, Shuai Guo, Zhili Li

**Affiliations:** 1 Department of Biophysics and Structural Biology, Institute of Basic Medical Sciences, Chinese Academy of Medical Sciences & School of Basic Medicine, Peking Union Medical College, Beijing 100005, P.R. China

## Abstract

Lipid composition in cell membrane is closely associated with cell characteristics. Here, matrix-assisted laser desorption/ionization- Fourier transform ion cyclotron resonance mass spectrometry was employed to *in situ* determine membrane components of human mammary epithelial cells (MCF-10 A) and six different breast cancer cell lines (*i.e.*, BT-20, MCF-7, SK-BR-3, MDA-MB-231, MDA-MB-157, and MDA-MB-361) without any lipid extraction and separation. Partial least-square discriminant analysis indicated that changes in the levels of these membrane lipids were closely correlated with the types of breast cell lines. Elevated levels of polyunsaturated lipids in MCF-10 A cells relative to six breast cancer cells and in BT-20 cells relative to other breast cancer cell lines were detected. The Western blotting assays indicated that the expression of five lipogenesis-related enzymes (*i.e.*, fatty acid synthase 1(FASN1), stearoyl-CoA desaturase 1 (SCD1), stearoyl-CoA desaturase 5 (SCD5), choline kinase α (CKα), and sphingomyelin synthase 1) was associated with the types of the breast cells, and that the SCD1 level in MCF-7 cells was significantly increased relative to other breast cell lines. Our findings suggest that elevated expression levels of FASN1, SCD1, SCD5, and CKα may closely correlated with enhanced levels of saturated and monounsaturated lipids in breast cancer cell lines.

Tumor growth leads to an increased “rate” of “*de novo*” synthesis and turnover of membrane lipids, and thousands of these lipid molecules play important roles in biological process such as the signaling and metabolic pathways^1^,^2^. Alterations in membrane lipid components can change the activity of membrane proteins that serve as ion channels, transporters, receptors, signal transducers, and enzyme activity^3^ and deeply influence cellular functions^4^. The phenomena linked to changes in cell membrane are suspected to play important roles during cancer transformation. Therefore, the determination of the membrane lipid components of tumor cells is essential in understanding tumor biological properties. Three main lipid species exist in mammalian cells, including glycerol-based lipids, cholesterol, and ceramide-based sphingolipids. Moreover, substantial studies in human cancers, such as colorectal^5^, lung^6^, liver^7^, and breast cancers^8^,^9^,^10^, have shown that changes in lipid levels may be involved in the onset of cancer and its progression.

At present, phospholipid-based study may be helpful for the clinical diagnosis of breast cancer^11^. Phosphatidylcholines (PCs), one major component of lipids in cell membrane, contribute to proliferative growth in cancer cells since the synthesis was increased in response to *de novo* fatty acids (FAs) synthesis^12^. Elevated levels of alkylacyl PCs were detected in malignant breast cancer cells relative to non-malignant cells^8^. It was found that phosphatidylethanolamine (PE) played an important role in the process of cell division, especially during cytokinesis^13^. Elevated levels of monounsaturated PEs (*e.g.*, PE(O-16:00/18:01) and PE(O-18:00/18:01)) were observed in the mouse aggressive breast cancer cell line^10^, and phosphatidylinositols (PIs), which are located on the cytoplasmic face of the membrane, participate in cell signaling and vesicle formation^2^. Decreased level of PI(18:0/18:1) in non-malignant cells relative to human breast cancer cell lines was also detected^8^. Sphingomyelins (SMs) were closely related to cell membrane fluidity^14^, and SMs and their metabolites also influenced the survival and migration of cancer cells^15^. Previous study has indicated that the ratio of total FAs to total unsaturated FAs was closely correlated with metastatic ability^9^.

Recently, mass spectrometry-based approach for lipidomic analysis has provided a new platform for quantitative, qualitative, and functional studies of individual lipid species in cells, tissues, or biological fluids^16^, and plant metabolism 17. Especially, matrix-assisted laser desorption/ionization (MALDI) mass spectrometry has shown the capability to *in situ* detect rapidly multiple cancer-specific lipid changes^18^. Recently, several MALDI matrices have been applied to the detection of small molecules, such as 1, 8-bis(dimethylamino)naphthalene (DMAN) for FAs analysis^19^ and 2,5-dihydroxybenzoic acid (DHB) for PCs and SMs analysis.

In this study, we have directly performed membrane lipid profiling of one human mammary epithelial MCF-10A cells and six different breast cancer cell lines (*i.e.*, BT-20, MCF-7, SK-BR-3, MDA-MB-231, MDA-MB-157, and MDA-MB-361) using MALDI-Fourier transform ion cyclotron resonance mass spectrometry (FTICR MS) to investigate the associations of membrane lipid components with their metastatic ability. Our data indicate that differences in the levels of the membrane lipids, such as PCs, SMs, FAs, PEs, and PIs, could be used to differentiate the above-mentioned seven cell lines, and that the unsaturated degree of these lipids might be correlated with the types of breast cancer cells.

## Results

### Differences in lipid levels of seven different breast cell lines

**Figure 1:**
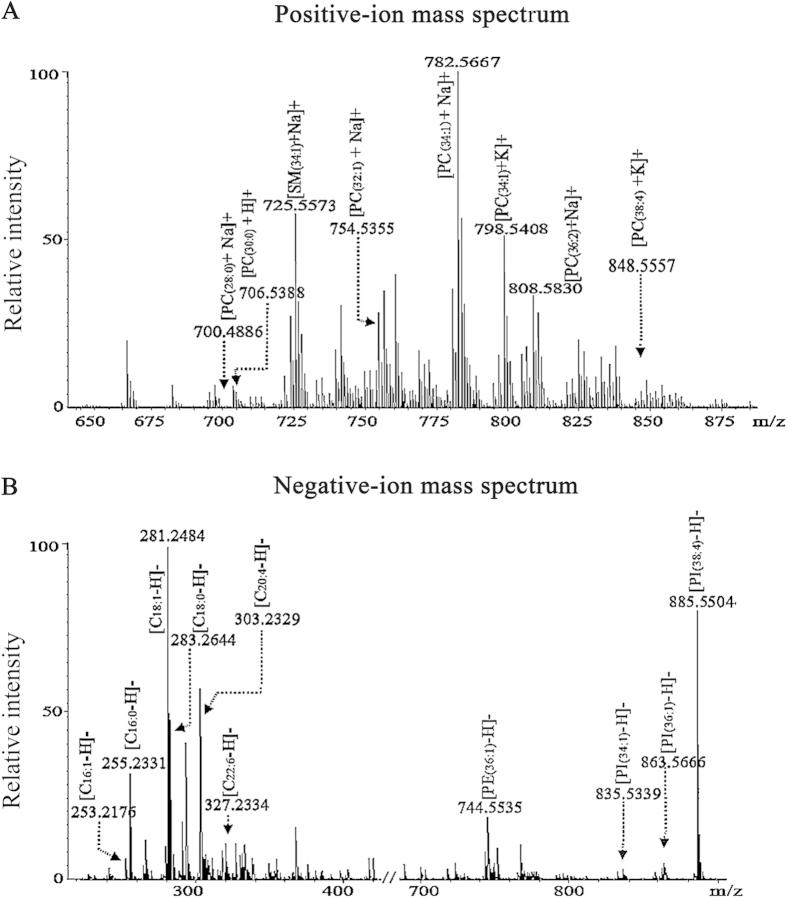
Representative mass spectra of MCF-10 A cells in the positive (**A**) and negative (**B**) ion modes.

**Figure 2:**
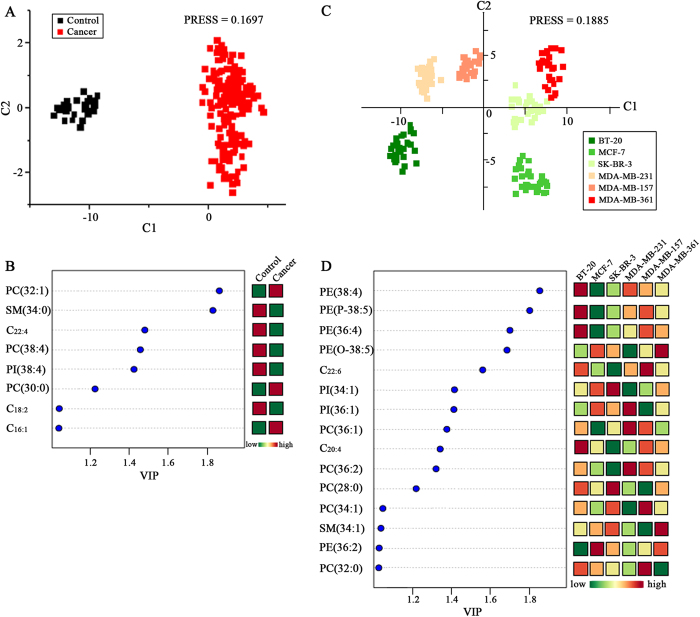
PLS-DA score plots of membrane lipids detected in this study between MCF-10A cells and breast cancer cell lines. (**A**) PLS-DA model analysis of 180 common metabolites to differentiate six different breast cancer cell lines (*i.e.*, BT-20, MCF-7, SK-BR-3, MDA-MB-231, MDA-BR-157, and MDA-BR-361) from MCF-10A control. (**B**) Significantly changed levels of 8 common lipids (*i.e.*, PC(32:1), SM(34:0), C_22:4_, PC(38:4), PI(38:4), PC(30:0), C_18:2_, and C_16:1_) with the VIP values of >1.0 between MCF-10A and six different breast cancer cell lines. (**C**) PLS-DA model analysis of the 180 common metabolites from six different breast cancer cell lines to differentiate six breast cancer cell lines. (**D**) Significantly changed levels of 15 common lipids (*i.e.,* PE(38:4), PE(P-38:5), PE(36:4), PE(O-38:5), C_22:6_, PI(34:1), PI(36:1), PC(36:1), C_20:4_, PC(36:2), PC(28:0), PC(34:1), SM(34:1), PE(36:2), and PC(32:0)) with the VIP values of >1.0 between six breast cancer cell lines.

In this study, an untargeted membrane lipid profiling was performed to *in situ* analyze intact cell samples using MALDI-FTICR MS platform. Representative mass spectra of MCF-10 A cells are shown in Fig. 1. Finally, a total of 180 membrane lipids including 69 detected in the positive ion mode and 111 detected in the negative ion mode were observed in seven different breast cell lines, and the detailed information on their *m/z* values are listed in Supplementary Table S1. PLS-DA score plot revealed the obvious differences between control (MCF-10A) and six different breast cancer cell lines (BT-20, MCF-7, SK-BR-3, MDA-MB-231, MDA-MB-157, and MDA-MB-361), with the predicted residual sum of square (PRESS) of 0.1697 (Fig. 2A), and 46 lipids have the variable importance in the projection (VIP) values of >1.0 (Supplementary Table S2). It should be noted that 8 simultaneously up- or down-regulated lipids in breast cancer cells relative to control were observed, and they are PC(32:1), SM(34:0), C_22:4_, PC(38:4), PI(38:4), PC(30:0), C_18:2_, and C_16:1_. The detailed information on their identification is shown in Supplementary Fig. S1 and Table S3. As shown in Fig. 2B, the levels of PC(32:1), PC(30:0), and C_16:1_ in breast cancer cells were significantly up-regulated, while the levels of SM(34:0), PC(38:4), PI(38:4), C_18:2_, and C_22:4_ in breast cancer cells were remarkably down-regulated relative to control. Their detailed change trends are shown in Supplementary Fig. S2A.

PLS-DA core plot of 180 common membrane lipids from six different breast cancer cell lines could generate six obvious clusters, with the PRESS of 0.1885 (Fig. 2C). BT-20 (ER-/PR-) cells derived from a primary tumor^20^ was located in the lower left hand quadrant, MCF-7 (ER+/PR+) cells from a metastatic tumor with a weakly invasive ability was in the lower right hand quadrant, highly invasive MDA-MB-231 (ER-/PR-) cells^21^,^22^ and MDA-MB-157 (ER-/PR-) cells^23^ derived from metastatic pleural effusions of human breast carcinoma were found in the upper left hand quadrant, and MDA-MB-361 (ER+/PR+) cells from a brain metastasis with weakly luminal epithelial-like phenotype^24^ were found in the upper right hand quadrant. Interestingly, SK-BR-3 (ER-/PR-) cells with weakly luminal epithelial-like phenotype^20^ were located at the right side of horizontal axis. It was found that a total of 49 lipids with the VIP values of >1.0 have contributed to characterize their differences. The detailed information on their identification is shown in Supplementary Table S3. Most importantly, 15 important lipids which mainly contributed to differentiate six different breast cancer cell lines were selected, and they are PE(38:4), PE(P-38:5), PE(36:4), PE(O-38:5), C_22:6_, PI(34:1), PI(36:1), PC(36:1), C_20:4_, PC(36:2), PC(28:0), PC(34:1), SM(34:1), PE(36:2), and PC(32:0). As shown in Fig. 2D, the levels of PE(38:4), PE(P-38:5), PE(36:4), and C_20:4_ in BT-20 cells relative to other five breast cancer cells were significantly increased, decreased levels of PE(38:4), PE(P-38:5), PE(36:4), and PC(36:1) in MCF-7 cells relative to other five cancer cells were obviously observed, the levels of PI(34:1) and PC(28:0) in SK-BR-3 relative to other five cancer cells were significantly increased, the levels of PI(36:1), PC(36:1), and PC(36:2) in MDA-MB-231 relative to other five cancer cells were obviously increased, the levels of PI(36:1), PC(28:0), and SM(34:1) in MDA-BR-157 relative to other five cancer cells were significantly decreased, and the levels of PE(O-38:5) and SM(34:1) in MDA-MB-361 relative to other five cancer cells were significantly increased. Their detailed change trends are shown in Supplementary Fig. S2B, and the *p* values of the above-mentioned 15 lipids between six different breast cancer cell lines are listed in Supplementary Table S4.

### Correlation and cluster analysis of characteristic lipids in seven different breast cancer cells

**Figure 3:**
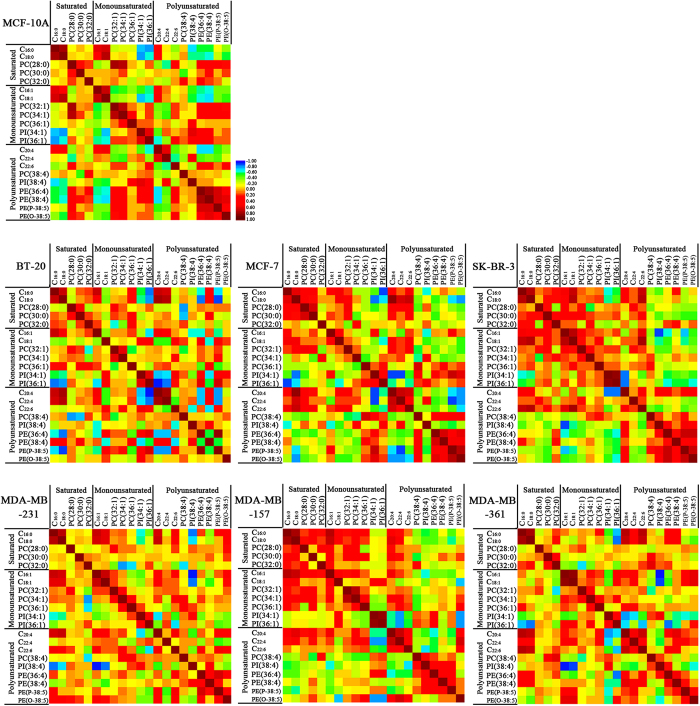
Correlation between membrane lipids between seven breast cell lines. Red, positive correlation; blue, negative correlation.

To confirm further the associations between the levels of the membrane lipids with specific fatty acyl chains and saturation with different breast cancer cells, Spearman correlation analysis was carried out to explore their relationships. Fig. 3 show the correlation coefficients (r) between lipid species highlighted in colors for different cell lines, and Spearman correlation analysis indicated that r >0.35 or <−0.35 indicate statistically significant difference (*p* < 0.05) between lipid species. The detailed information is listed in Supplementary Table S5-S11. For MCF-10 A cells as a control, saturated FAs (*i.e.,*C_16:0_ and C_18:0_) were positively correlated with monounsaturated FAs (*i.e.,*C_16:1_ and C_18:1_), and both were negatively correlated with unsaturated lipids (*i.e.,* PI(34:1), PI(36:1), PI (38:4), PE(36:4), PE(38:4), PE(P-38:5), and PE(O-38:5)). The latter four were strongly and positively correlated with PCs (*i.e.,* PC(28:0), PC(30:0), PC(32:1), and PC(34:1)) and C_22:6_. However, for BT-20, MDA-MB-231, and MDA-MB-361 cells, the above-mentioned correlations were not obviously observed. For MCF-7, SK-BR-3, and MDA-MB-157 cells, saturated and monounsaturated species (*i.e.*, C_16:0_, C_18:0_, PC(28:0), PC(30:0), PC(32:0), C_16:1_, C_18:1_, PC(32:1), PC(34:1), PC(36:1), PI(34:1), and PI(36:1)) were negatively correlated with polyunsaturated lipids (*i.e.*, PC(38:4), PI(38:4), PE(36:4), PE(38:4), PE(p-38:5), and PE(O-38:5)).

**Figure 4:**
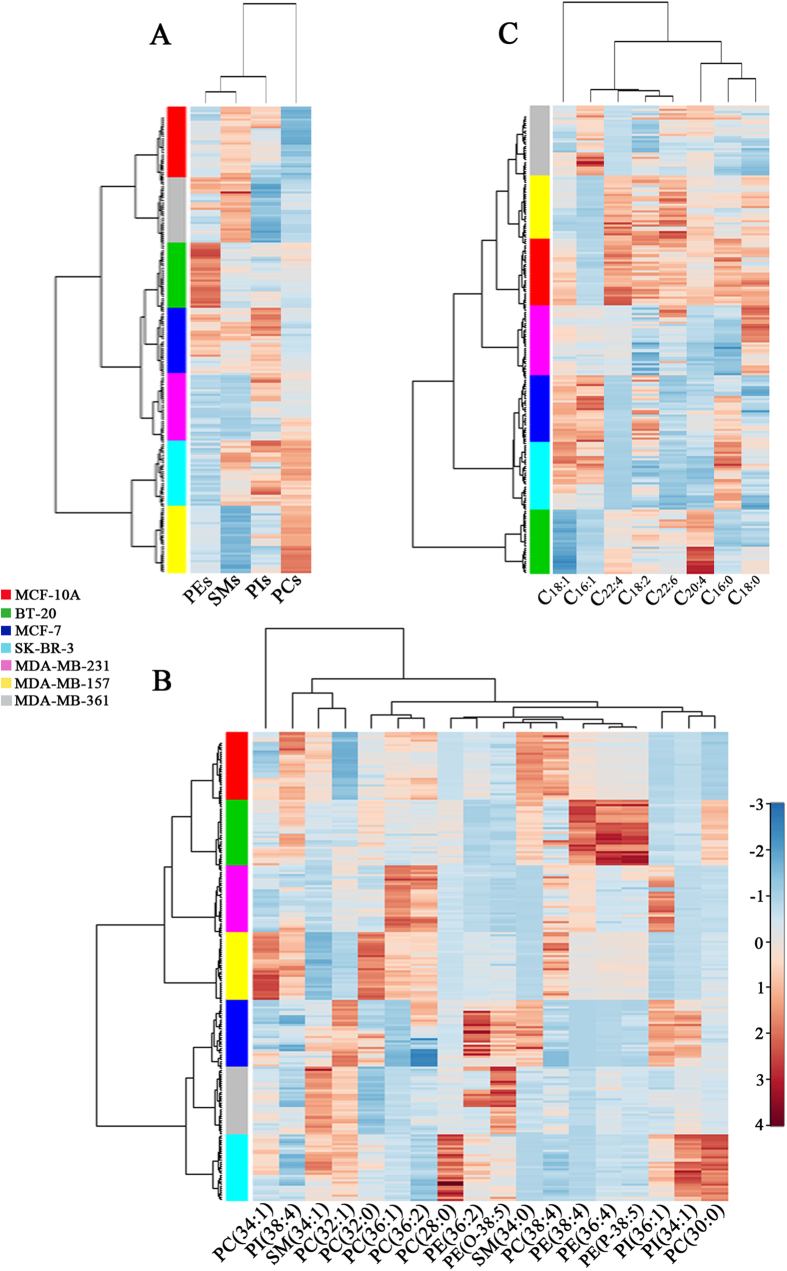
Hierarchical cluster analysis for membrane lipids. Heatmap of four kinds of lipid species (*i.e.*, PEs, SMs, PIs, and PCs) (**A**), of 18 important lipids (**B**), and of 8 fatty acids (**C**) in seven breast cell lines.

Hierarchical cluster analysis for the lipids of interest was described that used ‘Ward’ s linkage’ algorithms to arrange lipids according to similarity measure. As shown in Fig. 4A, a heatmap shows three clusters (*i.e.*, MCF-10A and MDA-MB-361; BT-20, MCF-7, and MDA-MB-231; SK-BR-3 and MDA-MB-157) based on the expression levels of four kinds of lipids (PEs, SMs, PIs, and PCs), suggesting that four kinds of lipid species have exhibited different biological behaviors in seven different breast cell lines. Based on 18 important species (*i.e.*, PC(34:1), PI(38:4), SM(34:1), PC(32:1), PC(32:0), PC(36:1), PC(36:2), PC(28:0), PE(36:2), PE(O-38:5), SM(34:0), PC(38:4), PE(38:4), PE(36:4), PE(P-38:5), PI(36:1), PI(34:1), and PC(30:0)), detailed cluster analysis has further showed obvious associations of the their expression levels with the types of breast cell lines (Fig. 4B). In addition, a heatmap of 8 FAs has indicated that their levels were also associated with the types of different breast cell lines (Fig. 4C).

### Association of expression levels of FASN1, SCD1, SCD5, CKα, and SMS1 with the types of breast cells

**Figure 5:**
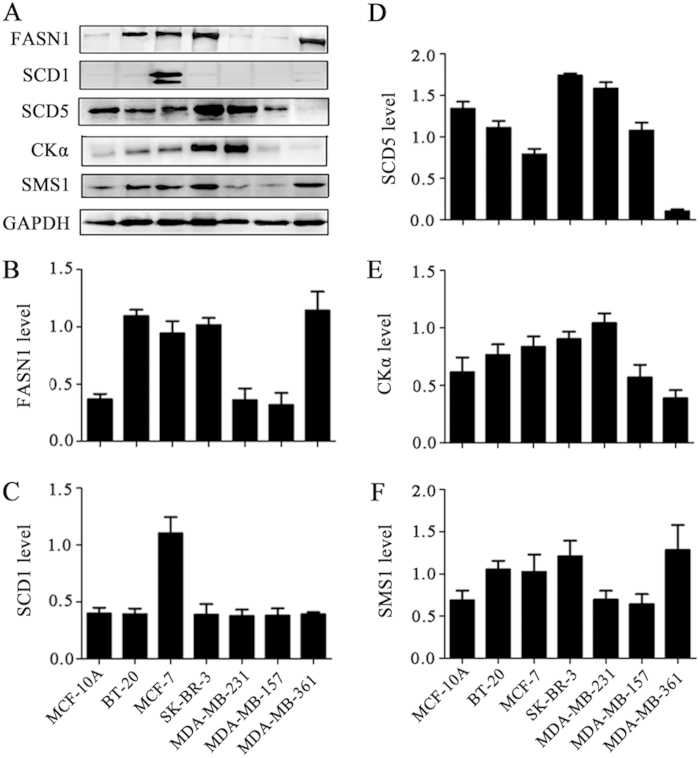
The expression levels of FASN1, SCD1, SCD5, CKα, and SMS1 in seven breast cell lines. (**A**) The optical images of FASN1, SCD1, SCD5, Ckα, SMS1, and GAPDH in the PVDF membranes. The expression levels of FASN1(**B**), SCD1 (**C**), SCD5 (**D**), CKα (**E**) and SMS1 (**F**) relative to the level of GAPDH in seven breast cell lines. The data are expressed as mean ± standard deviation (SD) in the triplicate, and statistically significant differences (*p* values) of these enzyme intensity between these breast cell lines are listed in Supplementary Table S12.

To determine the link of the expression levels of lipogenesis-related enzymes with the types of breast cells, the expression of FASN1, SCD1, SCD5, CKα, and SMS1 in seven different breast cell lines were detected using Western blotting. As shown in Fig. 5 and Supplementary Table S12, significantly increased levels of FASN1 in BT-20, MCF-7, SK-BR-3, and MDA-MB-361were detected relative to MCF-10A, MDA-MB-231, and MDA-MB-157. It should be noted that the SCD1 level in MCF-7 was significantly increased relative to other six breast cell lines (p < 0.001), while difference in the SCD1 level in MCF-10A, BT-20, SK-BR-3, MDA-BR-231, MDA-BR-157, and MDA-BR-361 was not detected (p > 0.5). The level of SCD5 in seven breast cell lines showed significant differences (p < 0.05), except between BT-20 and MDA-MB-157 (p > 0.09). Significant change in the CKα level in MCF-10A cells was detected relative to BT-20, MCF-7, SK-BR-3, MDA-MB-231, and MDA-MB-361 (p < 0.05), except MDA-MB-157 (p = 0.58), while significant increase in the CKα level in MDA-MB-231 cells was observed relative to MCF-10A, BT-20, MCF-7, MDA-MB-157, and MDA-MB-361 (p < 0.05), except SK-BR-3 (p = 0.09). The level of SMS1 in BT-20, MCF-7, SK-BR-3, and MDA-MB-361 was significantly up-regulated relative to MCF-10A, while significantly decreased level of SMS1in MDA-MB-231 and MDA-MB-157 was observed relative to other four breast cancer cells (p < 0.05).

## Discussion

Conventional lipidomic analytical approaches usually involve a complex sample preparation, including lipid extraction, separation by chromatography followed by mass spectrometric detection. In this study, mass spectrometry profiling was performed to *in situ* detect membrane lipids of seven breast cell lines without any lipid extraction and separation. It was found that direct biological analysis not only maintained cell integrity but also contributed to obtain rapidly lipid profiles. In addition, the application of FTICR MS could offer ultrahigh accurate mass measurements^25^. Finally, we have observed 180 common membrane lipids in seven different breast cell lines (Supplementary Table S1). In addition, 23 important lipids (*i. e*. PC(32:1), SM(34:0), C_22:4_, PC(38:4), PI(38:4), PC(30:0), C_18:2_, and C_16:1_, PE(38:4), PE(P-38:5), PE(36:4), PE(O-38:5), C_22:6_, PI(34:1), PI(36:1), PC(36:1), C_20:4_, PC(36:2), PC(28:0), PC(34:1), SM(34:1), PE(36:2), and PC(32:0 ) were again analyzed using electrospray ionization (ESI) technique, and most of them have similar change trends between MALDI and ESI. The detailed information is shown in Supplementary Fig. S2.

PLS-DA score plot has revealed that a combination of 8 membrane lipids (*i.e*., PC(32:1), SM(34:0), C_22:4_, PC(38:4), PI(38:4), PC(30:0), C_18:2_, and C_16:1_) could be used to obviously differentiate non-malignant human breast epithelial cells from six different breast cancer cell lines, suggesting that differences in membrane lipid components between non-malignant cells and malignant cells exist, and that significantly increased polyunsaturated lipids (*i.e.*, C_22:4_, PC(38:4), PI(38:4), and C_18:2_,) in the non-malignant cells relative to the malignant cells revealed the close link of the unsaturated degree of membrane lipids with the types of breast cancer cells. In addition, PLS-DA score plot of 180 lipids from six different breast cancer cell lines has remarkably classified them into six clusters (Fig. 2C), indicating that the membrane lipid components of six different breast cancer cells might also reflect differences in their membrane components. It is worth noting that the highest expression of polyunsaturated lipids (*i.e.*, PE(38:4), PE(P-38:5), PE(36:4), and C_20:4_) in BT-20 cell membrane from a primary tumor, along with a weakly invasive ability and negative for estrogen and progesterone receptors were observed relative to other five breast cancer cell lines derived from metastatic breast cancer tumors (Fig. 2D)^20^, indicating that the unsaturated degree of membrane lipids are closely associated with the types of breast cancer cells.

Substantial studies have indicated that FASN as a key enzyme in lipogenesis catalyzes *de novo* synthesis of palmitate (C_16:0_) from acetyl-CoA, and that the over-expression of FASN can suppress tumor necrosis factor-α production^26^ and contribute to poor prognosis in various types of cancer^27^,^28^. As shown in Fig. 5B, our data show increased level of FASN1 in BT-20, MCF-7, SK-BR-3, and MDA-MB-361 cells relative to MCF-10A, MDA-MB-231, and MDA-MB-157 was observed, but no correlation with its corresponding products, such as C_16:0_ and C_18:0_, was observed (Supplementary Fig. S3). One reason may be that free C_16:0_ and C_18:0_ in cells are usually located in cytoplasm, and they are difficultly detected by mass spectrometry and another reason may be that they are rapidly consumed to generate membrane lipids (*e.g.* PC(30:0) and PC(32:1)) or converted to other long-chain FAs by elongases and desaturases^29^, resulting in an increase in the levels of lipids (*e.g.* PC (34:1), PI(34:1) and PI(36:1)). Thus, the over-expression of FASN1 in cancer cells sustains an increasing demand for saturated and monounsaturated lipids during cell proliferation. It was found that the levels of polyunsaturated species (*e.g.,* PC(38:4) and PI(38:4) ) were down-regulated in most cancer cells. Some polyunsaturated FAs (*e.g.* C_22:6_ and C_20:5_) have been recognized as potential adjuvants against breast cancer cell proliferation, migration, and invasion^30^.

**Figure 6:**
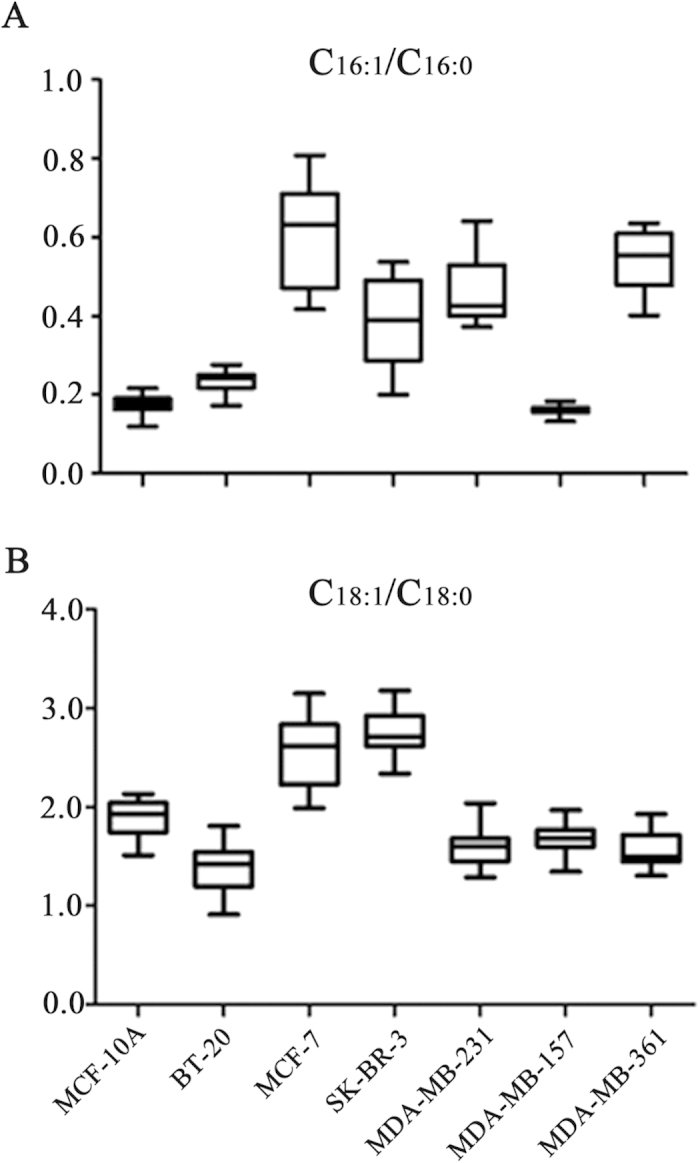
The level ratios of C_16:1_/C_16:0_ and C_18:1_/C_18:0_ in seven breast cell lines. All data are expressed as mean ± SD in the triplicate, and statistically significant differences (*p* values) of C_16:1_/C_16:0_ or C_18:1_/C_18:0_ between these breast cell lines are listed in Supplementary Table S4.

SCDs mainly catalyze the synthesis of C_16:1_ and C_18:1_ from C_16:0_ and C_18:0_, respectively. Previous studies have indicated that SCD1 (one isoform of SCD) played an important role in cancer progression^31^. In this study, it is found that high expression of SCD1 was detected in MCF-7 relative to BT-20, SK-BR-3, MDA-MB-231, MDA-MB-157, and MDA-MB-361 (Fig. 5C), which is in agreement with the high ratios of C_16:1_/C_16:0_ and C_18:1_/C_18:0_ (Fig. 6), and that significant increase in the SCD5 level (another isoform of SCD) in SK-BR-3 and MDA-MB-231cells might be another factor to increase the ratios of C_16:1_/C_16:0_ and/or C_18:1_/C_18:0_ , but we could not explain what caused the high ratio of C_16:1_/C_16:0_ in MDA-MB-361 cells (Fig. 6), which may be associated with other SCD enzymes.

PCs can be synthesized through cytidine diphosphate-choline pathway and choline kinase (CK) catalyzes the first step reaction in the choline pathway for *de novo* synthesis of PCs^32^. It was found that CKα, one isoform of CKs, was associated with the uncontrolled growth of cancer cells^33^. Our findings indicate that increased CKα level in BT-20, MCF-7, SK-BR-3, and MDA-MB-231 cells could lead to generate higher levels of PC(28:0), PC(30:0) and PC(32:1) relative to MCF-10 A cells (Supplementary Fig. S2). Specifically for MDA-MB-231 cells, it is found that the highest CKα level (Fig. 5E) was remarkably associated with the highest level of PC(36:1) and PC(36:2) relative to other six different breast cells. In the Golgi apparatus, SMS1 can utilize PC species as its substrate to produce SMs^34^. In this study, it is worth noting that the expression level of SMS1 in BT-20, MCF-7, SK-BR-3, and MDA-MB-361(Fig. 5F) was positively and closely correlated to change in the SM(34:1) level, specially for MDA-MB-361, but not related to the SM(34:0) level.

Gene microarray analysis is often used to be diagnostic biomarkers and distinguish metastatic ability of breast cancer cells^35^. Our results indicate that direct membrane lipid profiling coupled with the expression detection of lipogenesis-related enzymes may provide another insight to understand differences in molecular mechanisms of different breast cells, and to help distinguish different breast cell lines. For example, high expression of CKα, PC(36:1), and PC(36:2) might act as an useful biomarker panel to differentiate highly metastatic MDA-MB-231 cells from other breast cancer cells. Considering SM as a critical component of lipid rafts in mediating signal transduction^34^, increased levels of SMS1 and SM(34:1) in brain-metastasized breast cancer MDA-MB-361 cells may be involved in cell signaling as a characteristic feature. MCF-7 cells are characterized by the SCD1 expression, along with the low levels of PE(38:4), PE(P-38:5), PE(36:4), and PC(36:1). SK-BR-3 cells can express high levels of SCD5 enzyme and the highest level of PC(28:0), with the high intensity ratio of C_18:1_/C_18:0_ (Fig. 6). BT-20 cells can generate the high levels of PE(38:4), PE(P-38:5), and PE(36:4) relative to other breast cancer cells.

**Figure 7:**
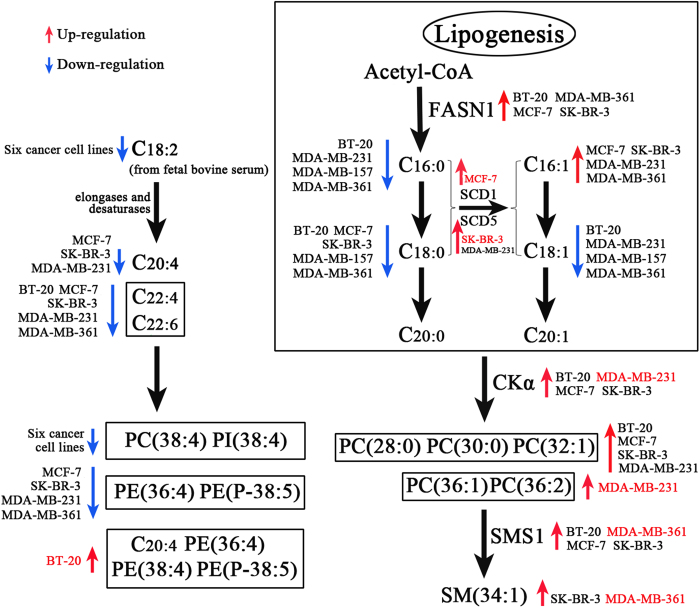
The correlations of membrane lipid levels with five lipogenesis-related enzymes. Arrow highlighted in red represents increased levels of the enzymes or lipids. Arrow highlighted in blue indicates decreased levels of the enzymes or lipids. Names of cell lines highlighted in red represent the highest increase in the levels of the enzymes or lipids. The detailed *p* values are listed in Supplementary Table S4 and S12.

Altogether, the correlations of changes in the levels of membrane lipids with the expression levels of five lipogenesis-related enzymes of six different breast cancer cell lines are shown in Fig. 7. It is found that polyunsaturated lipids in cancer cell membrane from a primary tumor, such as BT-20, were significantly increased relative to other five breast cancer cell lines from metastatic breast cancer tumors. It is worth noting that polyunsaturated lipids in breast cell membrane were really synthesized by themselves, not from exogenous fat, and that the unsaturated degree of membrane lipids for highly invasive breast cancer cells from metastatic breast cancer tumors was significantly decreased relative to healthy cells or breast cancer cells from a primary tumor. Most importantly, our results indicate that the membrane lipid phenotype and lipogenesis-related enzymes of breast cell lines might be associated with their malignancy. It should be noted that in this study some questions still remain unanswered, including the relationships between changes in the levels of membrane lipids, the lipogenesis-related protein expression, and cell properties for seven different cell lines. Further studies are need to determine these associations for each individual cell lines, such as by the addition of different exogenous lipids as cell food^36^.

## Conclusions

In this study, we have performed an *in situ* detection of membrane lipid profiles of seven different breast cell lines using an ultrahigh resolution MALDI-FTICR MS, without the requirement of separation and extraction process prior to mass spectrometric analysis. Our data show that a combination of 8 lipids could differentiate non-malignant MCF-10A cells from six different breast cancer cell lines and that a combination of 15 lipids could differentiate six different breast cancer cells. It is worth noting that significantly increased levels of monounsaturated lipids might be associated with the malignant degree of breast cancer cells. In addition, the presence of the high levels of polyunsaturated lipids in MCF-10A and BT-20 cell membranes relative to MCF-7, SK-BR-3, MDA-MB-231, MDA-MB-157, and MDA-MB-361 strongly suggest that polyunsaturated FAs were self-synthesized, not from dietary fat intake.

## Methods

### Cell culture

Human immortalized mammary epithelial cell line (MCF-10A) as a non-malignant control was obtained from Bioleaf (Shanghai, PR China). Human breast cancer cell lines including BT-20, MCF-7, SK-BR-3, MDA-MB-231, MDA-MB-157, and MDA-MB-361, were from the Cell Resource Center, Chinese Academy of Medical Sciences. MCF-10A and BT-20 cells were grown in minimum essential medium, MCF-7 cells was cultured in Dulbecco’s modified Eagle’s medium, and other four cell lines were grown in Roswell Park Memorial Institute-1640 medium. All media were supplemented with 10% fetal bovine serum (FBS) and 1% antibiotics (100 U/mL penicillin and 100 μg/mL streptomycin). Two conductive indium tin oxide (ITO)-coated glass slides were placed at the bottom of each of 100-mm culture dishes and cells were seeded in these dishes^37^, followed by the incubation until 80% confluence. All experiments were performed in accordance with relevant guidelines and regulations.

### Cell sample preparation

Cell-coated ITO-glass slides were washed with phosphate buffer saline three times and then air-dried. Matrix sublimation deposition was performed as our own previous study^38^. Briefly, the cell-coated ITO-slide was mounted onto the bottom of the condenser using thermally conductive double-sided adhesive tape and the slide was kept at approximately 4 °C using a water cooling system. 10 mg of DHB (Sigma-Aldrich, St. Louis, MO, USA) was sublimed under a fixed vacuum of 5 mTorr at an optimized sublimation temperature of 120 °C for 20 min. Finally, approximately 3.5 mg of DHB was coated at the surface of one cell-coated ITO-slide followed by mass spectrometric analysis in a positive ion mode. 20 mg of DMAN (Sigma-Aldrich, St. Louis, MO, USA) was sublimed under the above-mentioned vacuum condition at 50 °C for 9 min, and approximately 7.2 mg of DMAN was coated at the surface of another cell-coated ITO-slide from the same dish for mass spectrometric analysis in a negative ion mode. Each of the matrix-coated slide was placed at –20 °C for 30 min, and then the recrystallization was performed in a Petri dish (100 mm diameter × 15 mm depth) at a saturated solution atmosphere of methanol/water (1:1, v/v).

### Mass spectrometry profiling

All experiments were performed using a 9.4 T Apex-ultra^TM^ hybrid Qh-FTICR MS (Bruker Daltonics, Billerica, MA, USA) equipped with a 200 Hz, 355 nm Nd: YAG laser. All spectra were acquired using ApexControl 3.0.0 (Bruker Daltonics). A lipid mixture (PC(24:0) at *m/z* 622.44423, PC(32:0) at *m/z* 734.56943, PC(36:0) at *m/z* 790.63203, and PC(44:2) at *m/z* 898.72593 from Avanti Polar Lipids, Inc.) was used to calibrate the instrument over the *m/z* range of 600 ~ 1000 in the positive ion mode and a FA mixture of three commercially-available standard (C_16:0_ at *m/z* 255.23296, C_18:0_ at *m/z* 283.26425, and C_22:0_ at *m/z* 339.32684 from Sigma-Aldrich) combined with the ESI Tuning Mix (Part No. G2432A, Agilent Technology, Inc.) was used to calibrate the instrument over the *m/z* range of 100 ~ 1000 in the negative ion mode. For membrane lipid profiling, a mass spectrum was accumulated with three full scans once with 100 laser shots in the positive and negative ion modes, respectively. For each slide, eight mass spectra were randomly collected.

### Data processing and statistical analysis

Spectral data were processed with DataAnalysis Software 4.0 (Bruker Daltonics). Internal mass calibration was further performed against the known references ([PC(32:0) + H]^+^ at *m/z* 734.5694, [PC(32:0) + Na]^+^ at *m/z* 756.5514, [PC(34:1) + Na]^+^ at *m/z* 782.5670, and [PC(36:2) + Na]^+^ at *m/z* 824.5566) in the positive ion mass spectra and [C_18:0_-H]^−^ at *m/z* 283.2643, [C_22:6_-H]^−^ at *m/z* 327.2330, [PE(38:4) - H]^−^ at *m/z* 750.5443, and [PI(38:4)−H]^−^ at *m/z* 885.5499 in the negative ion mass spectra. After isotope deconvolution, monoisotopic peaks and their respective corresponding intensities were obtained using DAssis software (Bruker Daltonics). Peaks were selected with a signal-to-noise ratio of >3, relative intensity of >0.1%, and absolute intensity of >10,000. The resulting peaks between different samples were aligned within a narrow mass tolerance window of ±0.001 Da as a single lipid (or one variable). The peaks from [M+H]^+^, [M+Na]^+^, and [M+K]^+^ ions in the positive ion mode were further combined as one variable. The peaks observed at least in two-thirds of seven cell lines were selected, and the half of the baseline strength in each spectrum was adopted as their intensities of missing lipids. Finally, the resulting datasets were exported to Microsoft Excel. The intensities of lipids (or variables) from each mass spectrum were normalized to a constant number of 1000. Resulting datasets in the positive and negative ion modes were further combined into one dataset before statistical analysis.

Partial least-square discriminant analysis (PLS-DA) was performed to evaluate differences between control and different breast cancer cell lines. Univariate analysis was performed using non-parametric Wilcoxon-Mann-Whitney test. Correlation and cluster analysis were performed to analyze the correlation between saturated and unsaturated lipids. A *p* value of <0.05 was considered as statistically significant. Statistical analyses were performed using SAS software (version 9.2, SAS Institute Inc.) and SPSS software (version 16.0, SPSS Inc., Chicago, IL).

### Identification of membrane lipids of interest

Significantly changed lipids were identified as our own previous studies^38^,^39^. Briefly, lipids were identified with the aid of the available databases (the Lipid maps (http://www.lipidmaps.org/) and the METLIN database (http://metlin.scripps.edu/)), as well as their observed accurate m/z values relative to theoretical vales of <2 ppm, isotopic abundance distribution relative to theoretical distribution of <5%, and tandem mass spectra.

### Western blotting

Cells were harvested and suspended in RIPA lysis buffer (Solarbio Science & Technology Co., Beijing, China) which contains an antiprotease cocktail. After centrifugation, the amount of proteins in the supernatant was determined using bicinchoninic acid protein assay. Five aliquots of total proteins (30 mg) from each cell line were isolated by SDS-PAGE followed by transferring onto a polyvinylidene fluoride microporous membrane, respectively. These membranes were blocked with 5% (w/v) non-fat powdered milk in Tris-buffered saline plus 0.1% Tween-20 for 2 h at room temperature, and then incubated overnight at 4 °C with CKα-, SMS1-, SCD1-, SCD5-, and FASN1-specific antibodies (Abcam, San Francisco, CA, USA), respectively. These membranes were assayed against glyceraldehyde-3-phosphate dehydrogenase (GAPDH) as loading control. Secondary goat anti-mouse (Southern Biotechnology Assoc., Asbach, Germany) or goat anti-rabbit (Abcam) antibodies were used at a dilution of 1:2000 with the incubation for 2 h at room temperature. Signals were detected by chemiluminescent HRP substrate (Millipore, Billerica, MA, USA) and images were acquired using ImageQuant^TM^ LAS 4000 mini (GE Healthcare Uppsala, Sweden). The chemiluminescent signals were quantified using Quantity One software (Bio-Rad Laboratories) and were normalized to GAPDH, respectively.

One-way analysis of variance (ANOVA) was performed to determine statistically significant differences between non-malignant control and six different breast cancer cell lines. A *p* value of <0.05 was considered as statistically significant.

## Additional Information

**How to cite this article**: He, M. *et al.* *In situ* characterizing membrane lipid phenotype of breast cancer cells using mass spectrometry profiling. *Sci. Rep.* **5**, 11298; doi: 10.1038/srep11298 (2015).

## Supplementary Material

Supplementary Information
