# Supplementary material for: In situ characterizing membrane lipid phenotype of breast cancer cells using mass spectrometry profiling
Source: Sci Rep. 2015 Jun 10;5:11298. doi: 10.1038/srep11298 (PMC4462148; doi:10.1038/srep11298)
Supplement: Supplementary Information [file srep11298-s1.pdf]

Supplementary Figures S1-S3 and Tables S1-S12

In situ characterizing membrane lipid phenotype of breast cancer cells using mass spectrometry profiling

Manwen He, Shuai Guo, Zhili Li*

Department of Biophysics and Structural Biology, Institute of Basic Medical Sciences, Chinese Academy of Medical Sciences & School of Basic Medicine, Peking Union Medical College, Beijing 100005, P. R. China

***Corresponding author: Zhili Li**, Department of Biophysics and Structural Biology, Institute of Basic Medical Sciences, Chinese Academy of Medical Sciences & School of Basic Medicine, Peking Union Medical College, 5 Dongdan San Tiao, Beijing 100005, P.R. China.

E-mail: lizhili@ibms.pumc.edu.cn

Tel/Fax: +86-10-69156479

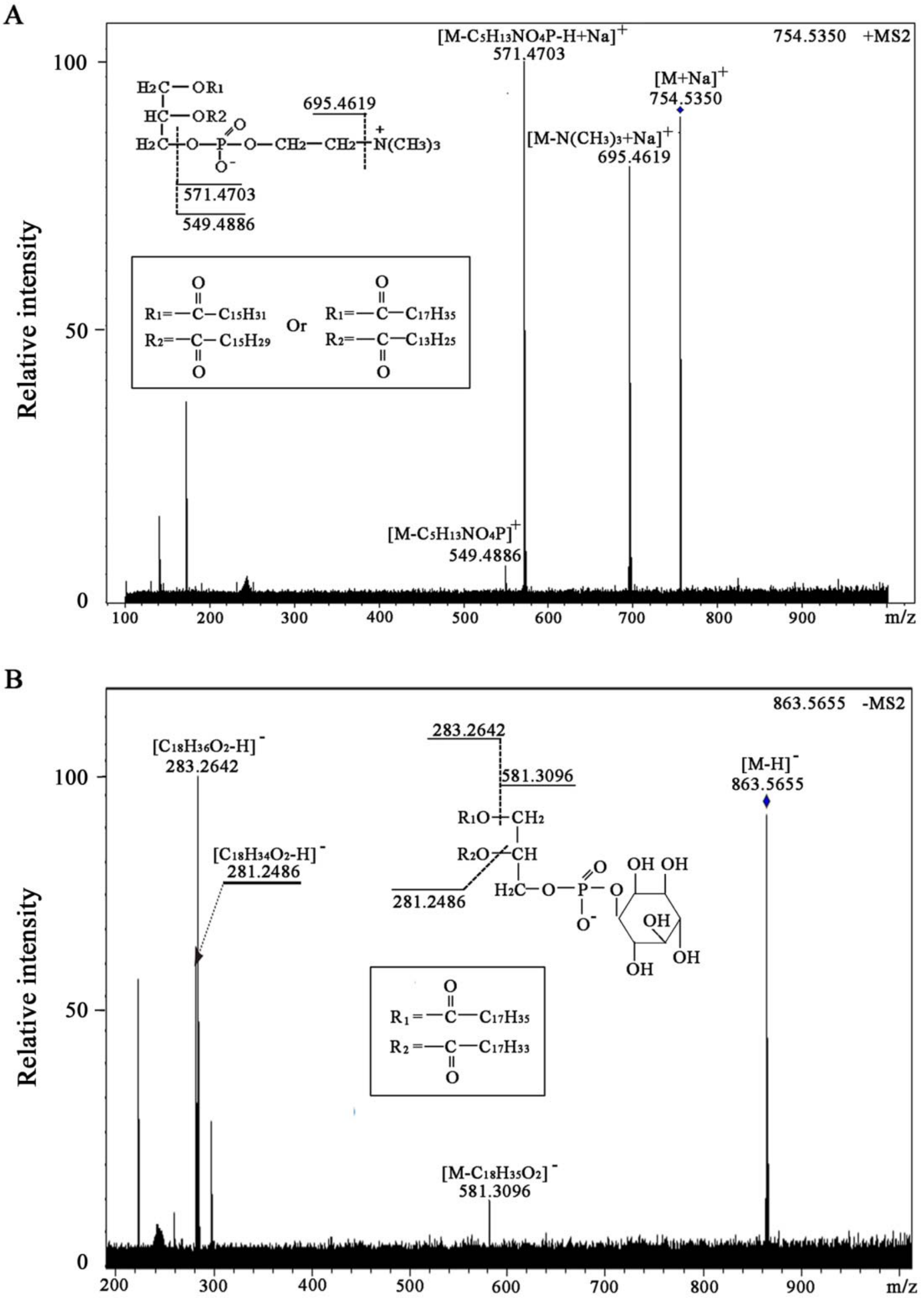

Supplementary Figure S1. Representative tandem mass spectra of the precursor ions $[PC(32:1) + Na]^+$ (A) and $[PI(18:0/18:1)-H]^-$ (B).

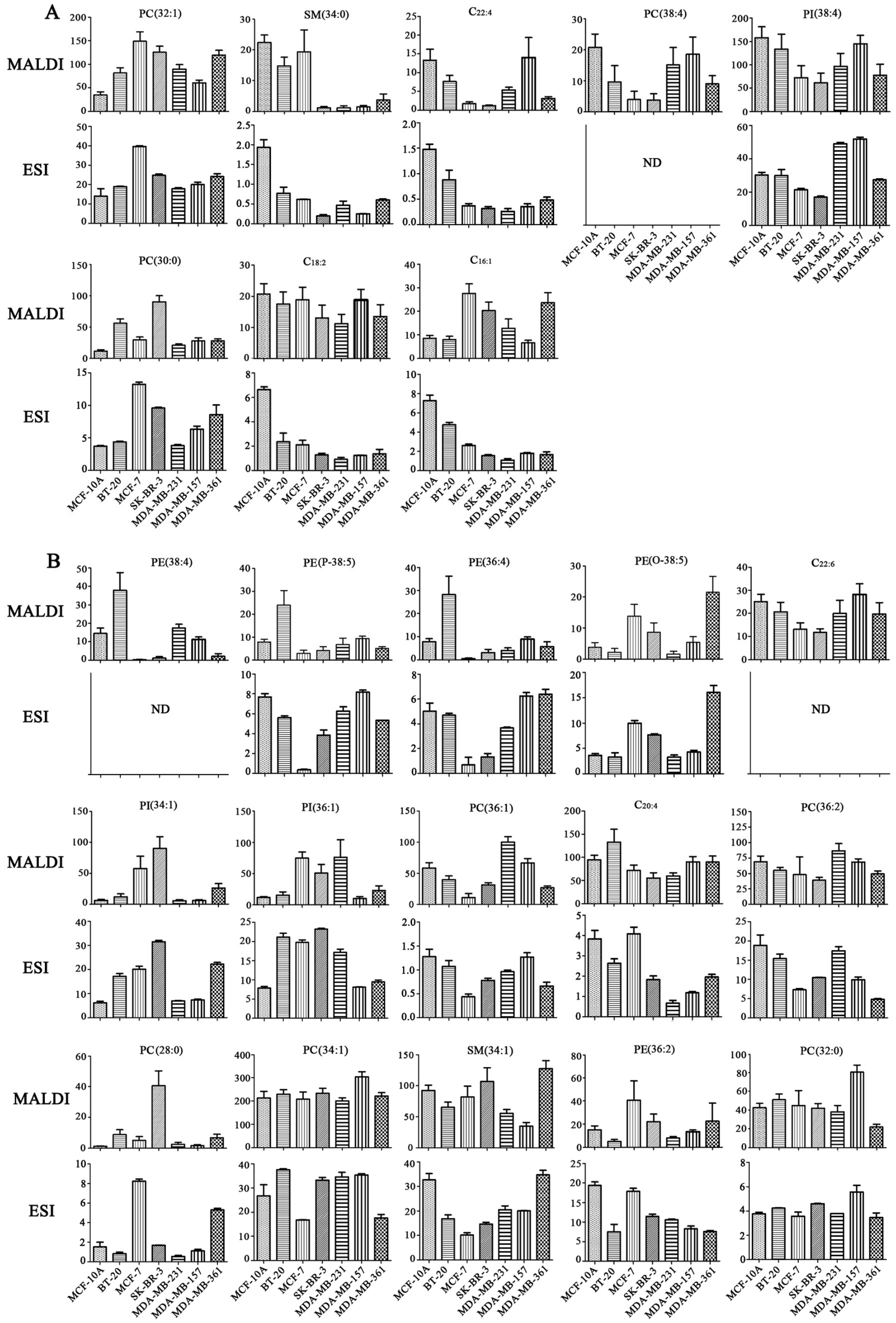

Supplementary Figure S2. Important membrane lipids. (A) 8 common membrane lipids (i.e., PC(32:1), SM(34:0), $C_{22:4}$, PC(38:4), PI(38:4), PC(30:0), $C_{18:2}$, and $C_{16:1}$) are used to differentiate malignant cancer cells from non-malignant cells (MALDI and ESI). (B) 15 important lipids (i.e., PE(38:4), PE(P-38:5), PE(36:4), PE(O-38:5), $C_{22:6}$, PI(34:1), PI(36:1), PC(36:1), $C_{20:4}$, PC(36:2), PC(28:0), PC(34:1), SM(34:1), PE(36:2), and PC(32:0)) are used to differentiate six different breast cancer cell lines (MALDI and ESI)**.** All data are expressed as mean ± SD and statistically significant differences (*p* values) of lipids between cell lines are listed in **Supplementary Table S4**.

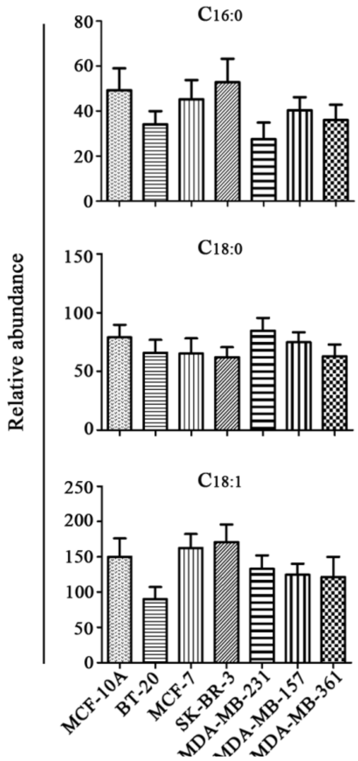

Supplementary Figure S3. The relative levels of $C_{16:0}$, $C1_{8:0}$, and $C_{18:1}$ in seven breast cell lines. Data are expressed as mean ± SD and statistically significant differences between breast cell lines are listed in **Supplementary Table S4**.

Supplementary Table S1. 180 lipids detected by mass spectrometry in the positive ion and negative ion modes

No.	m/z	No.	m/z	No.	m/z	No.	m/z	No.	m/z	No.	m/z
**Positive ion mode**						**Negative ion mode**					
1	621.5452	30#	760.5855	66	853.6555	70	253.2175	109	699.4977	148	808.5116
2	672.4218		782.5667	67	930.5451	71	255.2331	110	700.5345	149	810.5324
3#	678.5067		798.5408	68	932.5526	72	267.2331	111	701.5102	150	819.5255
	700.4886	31	762.5647	69	958.5735	73	269.2489	112	714.5112	151	824.4983
	716.4626	32	762.6009			74	279.2329	113	716.5272	152	829.4427
4	702.5426	33	770.5127			75	281.2484	114	722.5169	153	831.4592
5#	703.5750	34	770.5306			76	283.2644	115	723.494	154	833.5164
	725.5573	35	772.5760			77	301.2172	116	728.5658	155	835.5296
	741.5305	36	772.5466			78	303.2329	117	736.4917	156	837.5532
6	704.5226	37	774.6012			79	305.2487	118	736.5321	157	849.5517
7	705.5910	38	776.5802			80	307.2644	119	738.5104	158	855.4575
8#	706.5388	39	784.5844			81	309.2800	120	740.5261	159	857.4677
	728.5198	40	786.6013			82	327.2334	121	742.5405	160	857.5187
	744.4940	41	788.6167			83	329.2489	122	744.5536	161	859.5355
9	718.5379	42	787.6695			84	331.2644	123	746.5163	162	861.5532
10	718.5740	43	788.6169			85	339.2075	124	747.5244	163	863.5665
11	720.5538	44	790.5360			86	357.2803	125	748.5307	164	865.5069
12	720.5904	45	790.5598			87	359.2958	126	750.5451	165	869.5590
13	723.4942	46	792.5748			88	391.2259	127	752.5568	166	873.5605
14	724.5281	47	796.5822			89	403.2621	128	762.5084	167	875.5762
15	727.5724	48	804.5508			90	405.2788	129	764.5233	168	881.5090
16	730.5740	49	804.6110			91	417.2411	130	766.5416	169	883.5322
17#	732.5541	50	806.5666			92	418.2727	131	768.5607	170	885.5504
	754.5355	51#	808.5831			93	419.2568	132	770.5696	171	887.5639
	770.5092		824.5565			94	436.2834	133	772.5295	172	889.5806
18#	734.5700	52	810.5986			95	446.3052	134	773.5338	173	891.5992
	756.5514	53	813.6844			96	462.2993	135	774.5445	174	901.5938
	772.5257	54	814.5355			97	464.3162	136	775.5574	175	905.4932
19	739.4673	55	815.6991			98	480.3094	137	776.563	176	907.5482
20	739.4885	56	826.5930			99	546.2842	138	778.5758	177	909.5477
21	744.5140	57	828.5507			100	599.3212	139	790.5337	178	909.5508
22	744.5538	58	830.5309			101	601.2797	140	790.5428	179	911.5661
23	746.5702	59	830.5674			102	642.4912	141	792.5575	180	913.5800
24	746.6063	60	832.5831			103	671.4689	142	794.5746		
25	748.5492	61	834.5386			104	671.4762	143	799.6712		
26	748.5852	62	834.5991			105	673.4814	144	800.5067		
27	753.5880	63	837.6815			106	687.4986	145	802.5188		
28	758.5097	64	841.5704			107	687.5453	146	806.4927		
29	758.5698	65	848.5557			108	688.4967	147	807.5051		

'#': the different ion types such as $[M+H]^+$, $[M+Na]^+$, and $[M+K]^+$ were merged as one variable.

Supplementary Table S2. The lipids (or variables) with the VIP values of >1.0 between seven cell lines

No.	m/z	Control vs. Cancers VIP	m/z	Cancer vs. Cancer VIP
1	703.5750	1.132449	678.5067	1.202073
2	706.5388	1.222443	702.5426	1.258582
3	718.5379	1.174035	703.5750	1.081246
4	720.5904	1.09004	705.5910	2.499627
5	723.4942	1.485572	706.5388	1.489541
6	727.5724	1.817452	718.5740	1.766322
7	730.5740	1.265846	720.5538	1.160361
8	732.5541	1.853939	720.5904	1.370933
9	762.6009	1.201091	724.5281	1.244854
10	772.5760	1.035473	734.5700	1.010523
11	774.6012	1.166244	746.6063	1.050331
12	784.5844	1.431998	760.5855	1.089001
13	815.6991	1.557829	762.6009	1.002159
14	828.5507	1.032443	774.6012	1.510326
15	848.5557	1.463224	786.6013	1.321271
16	253.2175	1.028571	787.6695	2.283294
17	269.2489	1.287353	788.6167	1.384713
18	279.2329	1.075989	804.5508	1.408688
19	305.2487	1.464242	813.6844	2.58425
20	329.2489	1.128685	815.6991	2.31890
21	331.2644	1.484868	832.5831	1.342362
22	357.2803	2.363451	301.2172	2.160432
23	359.2958	2.678868	303.2329	1.354127
24	417.2411	1.377801	307.2644	1.000368
25	546.2842	1.538504	327.2334	1.565823
26	601.2797	1.187716	357.2803	1.156011
27	642.4912	1.760316	480.3094	1.392444
28	687.4986	1.993407	642.4912	1.225258
29	699.4977	1.152267	687.5453	1.247478
30	701.5102	1.521104	722.5169	1.689237
31	723.4940	1.598873	736.5321	1.743296
32	744.5536	1.270499	742.5405	1.014173
33	747.5244	1.222801	744.5536	1.248841
34	772.5295	1.036459	748.5307	1.801054
35	775.5574	1.11949	750.5451	1.852431
36	778.5758	1.557483	752.5568	1.073956
37	794.5746	1.183277	762.5084	1.720026
38	799.6712	1.729596	764.5233	1.673396
39	831.4592	1.143957	766.5416	1.221532
40	833.5164	1.122948	799.6712	1.164897
41	855.4575	1.304461	808.5116	1.191248
42	857.4677	1.833081	831.4592	1.121104
43	857.5187	1.002441	835.5296	1.401503
44	859.5355	1.149364	863.5665	1.401274
45	881.5090	1.025686	865.5069	1.234659
46	885.5504	1.420065	869.5590	1.630006
47			873.5605	1.260766
48			901.5938	1.454521
49			909.5477	1.106243

Supplementary Table S3. Identification of significantly changed lipids using tandem mass spectrometry

Matched lipids [1]	Molecular Formula	Ion Form	Observed m/z	Theoretical m/z	Error (Da)	Isotope distribution M : (M+1)	Fragments	
						Observed / Theoretical	Observed m/z	Theoretical m/z
PC(28:0)	$C_{36}H_{72}NO_8P$	$[M+H]^+$	678.5067	678.5068	-0.0001	100.00:37.02/100.00:40.45	678.5073/184.0733	678.5068/184.0733
		$[M+Na]^+$	700.4886	700.4888	-0.0002	100.00:37.84/100.00:40.44	700.4890/641.4150/517.4229/495.4407	700.4888/641.4153/517.4227/495.4408
		$[M+K]^+$	716.4626	716.4627	-0.0001	100.00:42.50/100.00:40.45	716.4619/657.3889/533.3973	716.4627/657.3892/533.3967
PC(30:0)	$C_{38}H_{76}NO_8P$	$[M+H]^+$	706.5388	706.5381	0.0007	100.00:45.13/100.00:42.66	706.5382/184.0733	706.5381/184.0733
		$[M+Na]^+$	728.5198	728.5201	-0.0003	100.00:38.60/100.00:42.65	728.5202/669.4465/545.4541/523.4720	728.5201/669.4466/545.4540/523.4721
		$[M+K]^+$	744.4940	744.4940	0.0000	100.00:43.25/100.00:42.66	744.4920/685.4190/561.4278	744.4940/685.4205/561.4256
PC(32:0)	$C_{40}H_{80}NO_8P$	$[M+H]^+$	734.5700	734.5694	0.0006	100.00:40.44/100.00:44.87	734.5697/184.0734	734.5694/184.0733
		$[M+Na]^+$	756.5514	756.5514	0.0000	100.00:47.89/100.00:44.86	756.5511/697.4773/573.4859	756.5514/697.4779/573.4853
		$[M+K]^+$	772.5257	772.5253	0.0004	100.00:39.99/100.00:44.87	772.5254/713.4518/589.4592	772.5253/713.4518/589.4593
PC(32:1)	$C_{40}H_{78}NO_8P$	$[M+H]^+$	732.5541	732.5538	0.0003	100.00:43.97/100.00:44.85	732.5538/184.0733	732.5538/184.0733
		$[M+Na]^+$	754.5355	754.5357	-0.0002	100.00:43.79/100.00:44.83	754.5350/695.4619/571.4703/549.4886	754.5357/695.4622/571.4697/549.4877
		$[M+K]^+$	770.5092	770.5097	-0.0005	100.00:45.72/100.00:44.85	770.5089/711.4360/587.4442	770.5097/711.4362/587.4436
PC(36:1)	$C_{44}H_{86}NO_8P$	$[M+Na]^+$	810.5986	810.5983	0.0003	100.00:48.90/100.00:49.25	810.5980/751.5253/627.5321	810.5983/751.5248/627.5323
PC(36:2)	$C_{44}H_{84}NO_8P$	$[M+Na]^+$	808.5831	808.5827	0.0004	100.00:49.65/100.00:49.23	808.5819/749.5086/625.5175	808.5827/749.5092/625.5166
		$[M+K]^+$	824.5565	824.5566	-0.0001	100.00:50.50/100.00:49.24	824.5562/765.4835	824.5566/765.4831
PC(38:4)	$C_{46}H_{84}NO_8P$	$[M+K]^+$	848.5557	848.5566	-0.0009	100.00:49.87/100.00:51.41	848.5570/789.4836/665.4900	848.5566/789.4831/665.4906
SM (34:0)	$C_{39}H_{81}N_2O_6P$	$[M+Na]^+$	727.5724	727.5724	0.0000	100.00:40.65/100.00:44.08	727.5724/668.4989/544.5064	727.5724/668.4989/544.5064
SM (34:1)	$C_{39}H_{79}N_2O_6P$	$[M+H]^+$	703.5750	703.5749	0.0001	100.00:31.09/100.00:44.07	703.5748/184.0733	703.5749/184.0733
		$[M+Na]^+$	725.5573	725.5568	0.0005	100.00:45.99/100.00:44.06	725.5558/666.4829/542.4916	725.5568/666.4833/542.4908
		$[M+K]^+$	741.5305	741.5307	-0.0002	100.00:43.43/100.00:44.07	741.5300/682.4569/558.4653	741.5307/682.4572/558.4647
PC(34:1)	$C_{42}H_{82}NO_8P$	$[M+H]^+$	760.5855	760.5851	0.0004	100.00:46.47/100.00:47.06	760.5850/184.0734	760.5851/184.0733
		$[M+Na]^+$	782.5667	782.5670	-0.0003	100.00:48.59/100.00:47.04	782.5662/723.4933/599.5016	782.5670/723.4935/599.5010
		$[M+K]^+$	798.5408	798.5409	-0.0001	100.00:49.81/100.00:47.06	798.5402/739.4672/615.4755	798.5409/739.4675/615.4749
$C_{16:0}$	$C_{16}H_{32}O_2$	$[M-H]^-$	255.2331	255.2330	0.0001	100.00:16.22/100.00:17.74	N/A	
$C_{16:1}$	$C_{16}H_{30}O_2$	$[M-H]^-$	253.2175	253.2173	0.0002	100.00:20.41/100.00:17.72	N/A	
$C_{18:0}$	$C_{18}H_{36}O_2$	$[M-H]^-$	283.2644	283.2643	0.0001	100.00:15.79/100.00:19.95	N/A	
$C_{18:1}$	$C_{18}H_{34}O_2$	$[M-H]^-$	281.2484	281.2486	0.0002	100.00:21.80/100.00:19.92	N/A	
$C_{18:2}$	$C_{18}H_{32}O_2$	$[M-H]^-$	279.2329	279.2330	-0.0001	100.00:16.22/100.00:19.90	N/A	
$C_{20:4}$	$C_{20}H_{32}O_2$	$[M-H]^-$	303.2329	303.2330	-0.0001	100.00:17.55/100.00:22.06	N/A	
$C_{22:4}$	$C_{22}H_{36}O_2$	$[M-H]^-$	331.2644	331.2643	0.0001	100.00:20.56/100.00:24.27	N/A	
$C_{22:6}$	$C_{22}H_{32}O_2$	$[M-H]^-$	327.2334	327.2330	0.0004	100.00:20.09/100.00:24.23	N/A	
PE(36:2)	$C_{41}H_{78}NO_8P$	$[M-H]^-$	742.5405	742.5392	0.0013	100.00:43.24/100.00:45.90	742.5392/283.2642/281.2487/279.2329	742.5392/283.2643/281.2486/279.2330
PE(36:4)	$C_{41}H_{74}NO_7P$	$[M-H]^-$	722.5137	722.5130	0.0007	100.00:42.09/100.00:45.82	722.5130/303.2330/255.2330	722.5130/303.2330/255.2330
PE(38:4)	$C_{43}H_{78}NO_7P$	$[M-H]^-$	750.5451	750.5443	0.0008	100.00:44.68/100.00:48.03	750.5441/464.3149/331.2641/303.2330/283.2644	750.5443/464.3146/331.2643/303.2330/283.2643
PE(P-38:5)	$C_{43}H_{76}NO_7P$	$[M-H]^-$	748.5292	748.5287	0.0005	100.00:45.47/100.00:48.01	748.5287/303.2330/281.2486	748.5287/303.2330/281.2486
PE(O-38:5)	$C_{43}H_{76}NO_8P$	$[M-H]^-$	764.5233	764.5236	-0.0003	100.00:45.00/100.00:48.04	764.5236/303.2330/301.2173/283.2642/281.2487	764.5236/303.2330/301.2173/283.2643/281.2486
PI(34:1)	$C_{43}H_{81}O_{13}P$	$[M-H]^-$	835.5339	835.5342	-0.0003	100.00:44.22/100.00:47.92	835.5342/283.2642/281.2487/255.2330/253.2173	835.5342/283.2643/281.2486/255.2330/253.2173
PI(36:1)	$C_{45}H_{85}O_{13}P$	$[M-H]^-$	863.5665	863.5655	0.0010	100.00:48.96/100.00:50.13	863.5655/581.3096/283.2642/281.2486	863.5655/581.3096/283.2643/281.2486
PI(38:4)	$C_{47}H_{83}O_{13}P$	$[M-H]^-$	885.5504	885.5499	0.0005	100.00:48.19/100.00:52.27	885.5498/305.2482/303.2328/283.2643/281.2486	885.5498/305.2486/303.2330/283.2643/281.2486

[1]Lipids with the VIP values of > 1 were identified by METLIN database, along with accurate molecular masses, isotopic abundance distribution and tandem mass spectra.

N/A: lipids were not identified by tandem mass spectra.

Supplementary Table S4. The p values obtained from Wilcoxon-Mann-Whitney test between two cell lines

	PC(28:0)	PC(30:0)	PC(32:0)	PC(32:1)	PC(36:1)	PC(36:2)	PC(38:4)	SM(34:1)	SM(34:0)	PC(34:1)	$C_{18:2}$	$C_{20:4}$	$C_{22:4}$	$C_{22:6}$	PE(36:2)	PE(36:4)	PE(38:4)	PI(38:4)	PE(P-38:5)	PE(O-38:5)	PI(34:1)	PI(36:1)	$C_{16:1}/C_{16:0}$	$C_{18:1}/C_{18:0}$	$C_{16:0}$	$C_{16:1}$	$C_{18:0}$	$C_{18:1}$
MCF-10A vs. BT-20	6.86E-18	6.86E-18	6.83E-10	6.86E-18	6.50E-16	7.52E-13	7.44E-14	9.83E-18	4.63E-16	0.0125755	3.46E-04	9.67E-09	4.40E-10	2.68E-05	2.87E-11	5.32E-10	2.87E-11	0.001811	5.32E-10	6.51E-06	3.06E-09	3.06E-05	3.66E-09	7.04E-10	3.39E-07	0.151547	8.58E-06	7.04E-10
MCF-10A vs. MCF-7	1.17E-16	6.86E-18	0.699457	6.86E-18	6.86E-18	0.00271	6.86E-18	8.91E-04	3.99E-06	0.4003222	0.124151	1.37E-08	2.87E-11	3.18E-11	1.23E-09	2.87E-11	2.87E-11	2.87E-11	3.88E-11	2.87E-11	2.87E-11	2.87E-11	2.87E-11	2.33E-09	0.054609	2.87E-11	1.45E-04	0.027851
MCF-10A vs. SK-BR-3	6.86E-18	6.86E-18	0.192598	6.86E-18	6.86E-18	6.86E-18	6.86E-18	4.38E-04	6.86E-18	5.53E-04	3.80E-08	4.29E-11	2.87E-11	2.87E-11	1.05E-05	1.69E-10	2.87E-11	2.87E-11	1.23E-09	5.84E-10	2.87E-11	2.87E-11	3.88E-11	2.87E-11	0.198358	3.88E-11	6.28E-07	2.63E-04
MCF-10A vs. MDA-MB-231	4.58E-08	6.86E-18	2.03E-04	6.86E-18	6.86E-18	1.58E-10	6.92E-07	6.86E-18	6.86E-18	0.0478694	1.15E-10	2.87E-11	2.87E-11	2.32E-04	6.37E-11	4.29E-11	1.54E-04	4.00E-09	0.00596129	9.18E-07	0.045945	8.12E-09	2.87E-11	1.80E-07	8.49E-10	3.46E-04	0.035783	0.00326
MCF-10A vs. MDA-MB-157	7.30E-04	6.86E-18	6.86E-18	7.73E-18	1.02E-06	0.508095	0.010965	6.86E-18	6.86E-18	6.86E-18	0.054609	0.225389	0.636141	0.003108	0.032054	0.002689	5.27E-06	0.017299	6.07E-06	6.16E-05	0.183321	0.015323	0.003939538	2.06E-05	3.46E-04	3.66E-07	0.049261	1.38E-05
MCF-10A vs. MDA-MB-361	6.86E-18	6.86E-18	6.86E-18	6.86E-18	6.86E-18	2.77E-16	1.11E-17	6.53E-17	6.86E-18	0.192598	1.63E-08	0.143282	2.87E-11	1.93E-05	0.711669	1.24E-06	2.87E-11	4.28E-11	1.69E-10	2.87E-11	2.87E-11	1.62E-09	2.83E-09	1.11E-06	4.27E-06	4.24E-11	2.78E-06	5.12E-04
BT-20 vs. MCF-7	4.95E-08	6.86E-18	0.007477	7.73E-18	6.86E-18	0.539512	9.59E-11	9.51E-07	1.40E-06	4.75E-05	0.10707	3.18E-11	2.87E-11	1.02E-09	2.87E-11	4.29E-11	2.87E-11	1.35E-09	5.32E-10	2.87E-11	3.51E-11	2.87E-11	2.87E-11	2.87E-11	2.98E-06	2.87E-11	0.72272	2.05E-10
BT-20 vs. SK-BR-3	6.86E-18	6.86E-18	1.98E-10	2.26E-17	5.04E-11	2.13E-17	1.18E-12	1.65E-16	6.86E-18	0.1879313	2.92E-04	2.87E-11	2.87E-11	2.87E-11	2.87E-11	4.84E-10	2.87E-11	7.73E-10	4.84E-10	5.32E-10	2.87E-11	2.87E-11	2.10E-08	2.87E-11	1.94E-09	3.88E-11	0.287112	1.04E-10
BT-20 vs. MDA-MB-231	6.70E-15	6.86E-18	1.37E-12	3.20E-04	6.86E-18	6.86E-18	1.80E-07	7.73E-18	6.86E-18	3.31E-11	1.37E-08	2.87E-11	6.26E-08	0.151547	3.34E-09	4.40E-10	2.87E-11	3.21E-06	4.84E-10	0.0124696	9.31E-10	8.12E-09	2.87E-11	0.0014059	1.63E-04	3.26E-05	1.11E-07	2.79E-09
BT-20 vs. MDA-MB-157	2.26E-17	6.86E-18	6.86E-18	1.39E-16	6.86E-18	9.64E-16	1.29E-11	1.71E-08	6.86E-18	1.49E-17	0.064595	1.02E-09	1.02E-09	3.66E-07	2.87E-11	5.32E-10	2.87E-11	0.287112	5.32E-10	1.63E-08	4.37E-09	2.98E-06	5.23E-11	1.79E-06	1.93E-05	4.34E-04	2.68E-05	7.44E-09
BT-20 vs. MDA-MB-361	1.72E-04	6.86E-18	6.86E-18	8.21E-18	2.77E-16	2.17E-07	0.344937	6.86E-18	6.86E-18	0.0848053	9.50E-05	3.05E-09	3.17E-11	0.178496	2.87E-11	4.84E-10	2.87E-11	8.12E-09	5.31E-10	2.87E-11	8.48E-10	2.46E-04	2.83E-09	0.0184427	0.254947	4.24E-11	0.407705	5.82E-07
MCF-7 vs. SK-BR-3	6.86E-18	6.86E-18	0.740717	1.94E-07	6.86E-18	0.06982	1	3.02E-07	1.31E-16	1.69E-05	8.01E-06	1.13E-05	2.35E-05	0.064595	4.22E-05	1.27E-10	2.98E-06	0.064595	0.00147962	7.32E-07	2.11E-07	1.63E-08	1.02E-07	0.0428195	0.007451	2.76E-04	0.266582	0.135407
MCF-7 vs. MDA-MB-231	1.61E-07	1.09E-14	0.202183	9.26E-18	6.86E-18	3.02E-11	5.48E-17	8.72E-18	1.10E-16	0.9889994	2.29E-08	6.98E-05	2.87E-11	3.13E-07	2.87E-11	7.73E-10	2.87E-11	0.030887	6.80E-08	2.87E-11	2.87E-11	0.078519	2.23E-06	4.29E-11	6.81E-09	3.51E-11	1.69E-11	1.33E-06
MCF-7 vs. MDA-MB-157	7.47E-15	0.008981	2.77E-16	6.86E-18	6.86E-18	0.013328	6.86E-18	4.40E-14	1.47E-16	2.13E-17	0.917573	9.90E-07	2.87E-11	3.88E-11	2.87E-11	2.87E-11	2.87E-11	2.87E-11	2.87E-11	2.87E-11	2.87E-11	2.87E-11	2.87E-11	2.87E-11	0.008135	2.87E-11	5.71E-04	2.35E-10
MCF-7 vs. MDA-MB-361	3.95E-04	0.003545	4.33E-17	1.44E-10	8.72E-18	0.634307	6.59E-12	8.73E-17	9.12E-16	0.0012845	6.97E-06	1.79E-06	3.51E-11	2.10E-08	9.84E-06	1.69E-10	9.84E-06	0.625626	7.89E-07	2.28E-07	4.37E-09	2.87E-11	0.016565389	2.83E-09	8.93E-05	0.030144	0.449376	1.79E-06
SK-BR-3 vs. MDA-MB-231	6.86E-18	6.86E-18	5.39E-04	1.17E-16	6.86E-18	6.86E-18	1.79E-17	6.86E-18	0.516973	1.51E-10	0.089092	0.113665	2.87E-11	1.86E-10	2.87E-11	5.79E-05	2.87E-11	3.21E-06	2.98E-06	4.40E-10	2.87E-11	1.21E-05	0.009266594	2.87E-11	2.05E-10	3.71E-05	2.00E-14	1.15E-06
SK-BR-3 vs. MDA-MB-157	6.86E-18	6.86E-18	6.86E-18	6.86E-18	6.86E-18	6.86E-18	6.86E-18	7.73E-18	2.10E-05	1.04E-17	5.27E-06	2.05E-10	2.87E-11	2.87E-11	2.33E-09	5.77E-11	2.87E-11	2.87E-11	3.64E-10	1.07E-06	2.87E-11	2.87E-11	2.87E-11	2.87E-11	6.51E-06	2.87E-11	6.98E-06	3.24E-14
SK-BR-3 vs. MDA-MB-361	6.86E-18	6.86E-18	6.86E-18	0.005238	2.27E-09	1.98E-14	1.19E-13	8.45E-06	7.29E-11	0.0018332	0.64672	1.86E-10	2.87E-11	3.88E-11	0.054606	1.20E-07	0.006818	0.009673	3.27E-04	8.56E-11	2.87E-11	8.48E-10	1.00E-05	2.83E-09	3.80E-08	0.066561	0.722127	2.28E-07
MDA-MB-231 vs. MDA-MB-157	2.03E-04	6.03E-14	6.86E-18	9.83E-18	7.73E-18	1.07E-12	0.001518	1.11E-17	5.55E-06	6.86E-18	7.73E-10	1.39E-10	2.87E-11	3.45E-06	5.23E-11	2.87E-11	3.51E-11	2.71E-08	4.85E-04	1.94E-09	0.72272	8.12E-09	2.87E-11	0.0124696	4.88E-08	8.70E-08	3.54E-04	0.024625
MDA-MB-231 vs. MDA-MB-361	1.29E-14	7.33E-17	7.28E-18	1.25E-17	6.86E-18	6.86E-18	7.60E-09	6.86E-18	4.81E-11	2.37E-09	0.017297	1.15E-10	6.37E-11	0.756198	1.69E-10	7.47E-06	2.87E-11	0.006235	0.00358465	2.87E-11	2.87E-11	8.12E-09	0.001532023	0.7067198	2.20E-05	5.81E-09	1.82E-13	0.044356
MDA-MB-157 vs. MDA-MB-361	2.13E-17	0.276055	6.86E-18	6.86E-18	6.86E-18	2.01E-17	3.11E-16	6.86E-18	4.84E-07	6.86E-18	3.97E-06	0.778781	2.87E-11	3.13E-07	0.756198	1.77E-09	2.87E-11	7.03E-11	2.87E-11	2.87E-11	2.87E-11	7.04E-10	2.83E-09	0.0165654	0.002561	4.24E-11	3.15E-05	0.293852

P values labelled in red represent no statistical differences.

Supplementary Table S5. Correlation of characteristic lipids in MCF-10A cell line (correlation coefficient and *p* values were listed between every two lipids)

	$C_{16:0}$	$C_{18:0}$	PC(28:0)	PC(30:0)	PC(32:0)	SM(34:0)	$C_{16:1}$	$C_{18:1}$	PC(32:1)	PC(34:1)	PC(36:1)	SM(34:1)	PI(34:1)	PI(36:1)	$C_{20:4}$	$C_{22:4}$	$C_{22:6}$	PC(38:4)	PI(38:4)	PE(36:4)	PE(38:4)	PE(P-38:5)	PE(O-38:5)
**$C_{16:0}$**	1	0.85451	−0.13771	0.04338	−0.12481	0.36151	0.66719	0.71034	−0.204	−0.15239	−0.18042	−0.04294	−0.70768	−0.76552	0.38509	−0.07631	−0.30323	−0.02825	−0.68988	−0.54527	−0.53904	−0.14616	−0.32948
**$C_{16:0}$**		<.0001	0.468	0.8199	0.5111	0.0497	<.0001	<.0001	0.2796	0.4214	0.34	0.8218	<.0001	<.0001	0.0356	0.6886	0.1033	0.8822	<.0001	0.0018	0.0021	0.4409	0.0754
**$C_{18:0}$**	0.85451	1	−0.04917	−0.05228	−0.00912	0.23515	0.58888	0.83315	−0.09455	−0.04694	−0.11769	−0.01001	−0.77887	−0.6485	0.47097	−0.10612	−0.22136	0.0941	−0.57241	−0.58087	−0.63115	−0.23471	−0.52436
**$C_{18:0}$**	<.0001		0.7964	0.7838	0.9618	0.211	0.0006	<.0001	0.6192	0.8054	0.5357	0.9581	<.0001	0.0001	0.0086	0.5768	0.2398	0.6209	0.0009	0.0008	0.0002	0.2119	0.0029
**PC(28:0)**	−0.13771	−0.04917	1	0.28899	0.22625	−0.53726	−0.32058	−0.22358	0.85895	0.80601	0.04917	−0.63826	−0.1475	0.18843	−0.31212	−0.55284	0.37931	−0.08165	−0.21379	0.50745	0.42024	0.45362	0.28943
**PC(28:0)**	0.468	0.7964		0.1214	0.2293	0.0022	0.0841	0.235	<.0001	<.0001	0.7964	0.0001	0.4367	0.3187	0.0931	0.0015	0.0387	0.668	0.2566	0.0042	0.0208	0.0118	0.1208
**PC(30:0)**	0.04338	−0.05228	0.28899	1	0.03404	0.27119	0.02113	0.00378	0.07453	0.21335	−0.1426	−0.18799	−0.22803	−0.1475	0.04739	−0.01268	0.02158	−0.49321	−0.12792	0.11546	0.25028	0.03137	0.22625
**PC(30:0)**	0.8199	0.7838	0.1214		0.8583	0.1472	0.9117	0.9842	0.6955	0.2576	0.4522	0.3198	0.2255	0.4367	0.8036	0.947	0.9099	0.0056	0.5005	0.5435	0.1822	0.8693	0.2293
**PC(32:0)**	−0.12481	−0.00912	0.22625	0.03404	1	−0.04561	−0.02158	0.02825	0.14572	−0.00111	−0.27964	0.27341	−0.03537	0.04828	−0.24093	−0.29255	0.05184	0.50122	0.00601	0.33793	0.21513	0.11591	−0.1644
**PC(32:0)**	0.5111	0.9618	0.2293	0.8583		0.8109	0.9099	0.8822	0.4423	0.9953	0.1345	0.1438	0.8528	0.8	0.1996	0.1167	0.7856	0.0048	0.9749	0.0678	0.2536	0.5419	0.3853
**SM(34:0)**	0.36151	0.23515	−0.53726	0.27119	−0.04561	1	0.46207	0.31034	−0.60356	−0.60089	−0.41135	0.51813	−0.23159	−0.54483	0.46118	0.37798	−0.28053	−0.02425	−0.23648	−0.37753	−0.28498	−0.31168	−0.00823
**SM(34:0)**	0.0497	0.211	0.0022	0.1472	0.8109		0.0102	0.0951	0.0004	0.0004	0.0239	0.0034	0.2182	0.0019	0.0103	0.0395	0.1332	0.8988	0.2083	0.0397	0.1269	0.0936	0.9656
**$C_{16:1}$**	0.66719	0.58888	−0.32058	0.02113	−0.02158	0.46207	1	0.77175	−0.50478	−0.48432	−0.34861	0.30011	−0.45139	−0.5871	0.45406	0.14527	−0.13014	−0.02469	−0.45584	−0.55996	−0.60578	−0.38643	−0.38954
**$C_{16:1}$**	<.0001	0.0006	0.0841	0.9117	0.9099	0.0102		<.0001	0.0044	0.0067	0.059	0.1071	0.0123	0.0006	0.0117	0.4437	0.493	0.8969	0.0114	0.0013	0.0004	0.0349	0.0334
**$C_{18:1}$**	0.71034	0.83315	−0.22358	0.00378	0.02825	0.31034	0.77175	1	−0.33615	−0.28187	−0.1822	0.18754	−0.71123	−0.6307	0.6396	0.099	−0.19511	0.00601	−0.51635	−0.64894	−0.67697	−0.36819	−0.47631
**$C_{18:1}$**	<.0001	<.0001	0.235	0.9842	0.8822	0.0951	<.0001		0.0693	0.1313	0.3352	0.321	<.0001	0.0002	0.0001	0.6027	0.3015	0.9749	0.0035	0.0001	<.0001	0.0453	0.0078
**PC(32:1)**	−0.204	−0.09455	0.85895	0.07453	0.14572	−0.60356	−0.50478	−0.33615	1	0.80467	0.14705	−0.62981	−0.04694	0.22892	−0.36507	−0.44472	0.24983	0.01268	−0.06385	0.48788	0.44561	0.42558	0.29032
**PC(32:1)**	0.2796	0.6192	<.0001	0.6955	0.4423	0.0004	0.0044	0.0693		<.0001	0.4381	0.0002	0.8054	0.2237	0.0473	0.0138	0.183	0.947	0.7375	0.0062	0.0136	0.019	0.1196
**PC(34:1)**	−0.15239	−0.04694	0.80601	0.21335	−0.00111	−0.60089	−0.48432	−0.28187	0.80467	1	0.50567	−0.67608	−0.05673	0.23826	−0.33526	−0.46874	0.24049	−0.09366	−0.07453	0.43582	0.44071	0.38287	0.32191
**PC(34:1)**	0.4214	0.8054	<.0001	0.2576	0.9953	0.0004	0.0067	0.1313	<.0001		0.0044	<.0001	0.7659	0.2048	0.0701	0.009	0.2005	0.6225	0.6955	0.0161	0.0148	0.0368	0.0828
**PC(36:1)**	−0.18042	−0.11769	0.04917	−0.1426	−0.27964	−0.41135	−0.34861	−0.1822	0.14705	0.50567	1	−0.30901	0.19956	0.18398	−0.03226	0.06607	−0.1911	−0.00779	0.34905	−0.03092	0.02825	0.00423	0.03715
**PC(36:1)**	0.34	0.5357	0.7964	0.4522	0.1345	0.0239	0.059	0.3352	0.4381	0.0044		0.0966	0.2904	0.3304	0.8656	0.7287	0.3117	0.9674	0.0587	0.8711	0.8822	0.9823	0.8455
**SM(34:1)**	−0.04294	−0.01001	−0.63826	−0.18799	0.27341	0.51813	0.30011	0.18754	−0.62981	−0.67608	−0.30901	1	0.14527	−0.12836	0.18443	0.27564	0.01268	0.36329	0.08031	−0.19644	−0.25695	−0.41313	−0.32814
**SM(34:1)**	0.8218	0.9581	0.0001	0.3198	0.1438	0.0034	0.1071	0.321	0.0002	<.0001	0.0966		0.4437	0.499	0.3292	0.1404	0.947	0.0485	0.6731	0.2981	0.1705	0.0233	0.0767
**PI(34:1)**	−0.70768	−0.77887	−0.1475	−0.22803	−0.03537	−0.23159	−0.45139	−0.71123	−0.04694	−0.05673	0.19956	0.14527	1	0.68854	−0.43804	0.19733	0.00957	0.10345	0.67742	0.30901	0.32547	0.00556	0.17998
**PI(34:1)**	<.0001	<.0001	0.4367	0.2255	0.8528	0.2182	0.0123	<.0001	0.8054	0.7659	0.2904	0.4437		<.0001	0.0155	0.2959	0.96	0.5864	<.0001	0.0966	0.0792	0.9767	0.3413
**PI(36:1)**	−0.76552	−0.6485	0.18843	−0.1475	0.04828	−0.54483	−0.5871	−0.6307	0.22892	0.23826	0.18398	−0.12836	0.68854	1	−0.63826	−0.32013	0.38687	0.16129	0.58131	0.46518	0.4238	0.16618	0.1911
**PI(36:1)**	<.0001	0.0001	0.3187	0.4367	0.8	0.0019	0.0006	0.0002	0.2237	0.2048	0.3304	0.499	<.0001		0.0001	0.0846	0.0347	0.3945	0.0008	0.0096	0.0196	0.3801	0.3117
**$C_{20:4}$**	0.38509	0.47097	−0.31212	0.04739	−0.24093	0.46118	0.45406	0.6396	−0.36507	−0.33526	−0.03226	0.18443	−0.43804	−0.63826	1	0.66808	−0.40245	−0.26007	−0.15684	−0.66274	−0.67341	−0.46207	−0.32147
**$C_{20:4}$**	0.0356	0.0086	0.0931	0.8036	0.1996	0.0103	0.0117	0.0001	0.0473	0.0701	0.8656	0.3292	0.0155	0.0001		<.0001	0.0275	0.1652	0.4078	<.0001	<.0001	0.0102	0.0832
**$C_{22:4}$**	−0.07631	−0.10612	−0.55284	−0.01268	−0.29255	0.37798	0.14527	0.099	−0.44472	−0.46874	0.06607	0.27564	0.19733	−0.32013	0.66808	1	−0.55907	−0.31257	0.37041	−0.44783	−0.39889	−0.43715	−0.17731
**$C_{22:4}$**	0.6886	0.5768	0.0015	0.947	0.1167	0.0395	0.4437	0.6027	0.0138	0.009	0.7287	0.1404	0.2959	0.0846	<.0001		0.0013	0.0926	0.0439	0.0131	0.029	0.0157	0.3486
**$C_{22:6}$**	−0.30323	−0.22136	0.37931	0.02158	0.05184	−0.28053	−0.13014	−0.19511	0.24983	0.24049	−0.1911	0.01268	0.00957	0.38687	−0.40245	−0.55907	1	−0.0683	−0.28587	0.37041	0.33749	0.12925	0.35573
**$C_{22:6}$**	0.1033	0.2398	0.0387	0.9099	0.7856	0.1332	0.493	0.3015	0.183	0.2005	0.3117	0.947	0.96	0.0347	0.0275	0.0013		0.7199	0.1257	0.0439	0.0682	0.496	0.0537
**PC(38:4)**	−0.02825	0.0941	−0.08165	−0.49321	0.50122	−0.02425	−0.02469	0.00601	0.01268	−0.09366	−0.00779	0.36329	0.10345	0.16129	−0.26007	−0.31257	−0.0683	1	0.09277	0.01224	−0.04116	0.01357	−0.18665
**PC(38:4)**	0.8822	0.6209	0.668	0.0056	0.0048	0.8988	0.8969	0.9749	0.947	0.6225	0.9674	0.0485	0.5864	0.3945	0.1652	0.0926	0.7199		0.6258	0.9488	0.829	0.9433	0.3233
**PI(38:4)**	−0.68988	−0.57241	−0.21379	−0.12792	0.00601	−0.23648	−0.45584	−0.51635	−0.06385	−0.07453	0.34905	0.08031	0.67742	0.58131	−0.15684	0.37041	−0.28587	0.09277	1	0.09677	0.07186	−0.14349	−0.12436
**PI(38:4)**	<.0001	0.0009	0.2566	0.5005	0.9749	0.2083	0.0114	0.0035	0.7375	0.6955	0.0587	0.6731	<.0001	0.0008	0.4078	0.0439	0.1257	0.6258		0.6109	0.7059	0.4494	0.5126
**PE(36:4)**	−0.54527	−0.58087	0.50745	0.11546	0.33793	−0.37753	−0.55996	−0.64894	0.48788	0.43582	−0.03092	−0.19644	0.30901	0.46518	−0.66274	−0.44783	0.37041	0.01224	0.09677	1	0.89366	0.701	0.49188
**PE(36:4)**	0.0018	0.0008	0.0042	0.5435	0.0678	0.0397	0.0013	0.0001	0.0062	0.0161	0.8711	0.2981	0.0966	0.0096	<.0001	0.0131	0.0439	0.9488	0.6109		<.0001	<.0001	0.0058
**PE(38:4)**	−0.53904	−0.63115	0.42024	0.25028	0.21513	−0.28498	−0.60578	−0.67697	0.44561	0.44071	0.02825	−0.25695	0.32547	0.4238	−0.67341	−0.39889	0.33749	−0.04116	0.07186	0.89366	1	0.72992	0.69477
**PE(38:4)**	0.0021	0.0002	0.0208	0.1822	0.2536	0.1269	0.0004	<.0001	0.0136	0.0148	0.8822	0.1705	0.0792	0.0196	<.0001	0.029	0.0682	0.829	0.7059	<.0001		<.0001	<.0001
**PE(P-38:5)**	−0.14616	−0.23471	0.45362	0.03137	0.11591	−0.31168	−0.38643	−0.36819	0.42558	0.38287	0.00423	−0.41313	0.00556	0.16618	−0.46207	−0.43715	0.12925	0.01357	−0.14349	0.701	0.72992	1	0.56796
**PE(P-38:5)**	0.4409	0.2119	0.0118	0.8693	0.5419	0.0936	0.0349	0.0453	0.019	0.0368	0.9823	0.0233	0.9767	0.3801	0.0102	0.0157	0.496	0.9433	0.4494	<.0001	<.0001		0.0011
**PE(O-38:5)**	−0.32948	−0.52436	0.28943	0.22625	−0.1644	−0.00823	−0.38954	−0.47631	0.29032	0.32191	0.03715	−0.32814	0.17998	0.1911	−0.32147	−0.17731	0.35573	−0.18665	−0.12436	0.49188	0.69477	0.56796	1
**PE(O-38:5)**	0.0754	0.0029	0.1208	0.2293	0.3853	0.9656	0.0334	0.0078	0.1196	0.0828	0.8455	0.0767	0.3413	0.3117	0.0832	0.3486	0.0537	0.3233	0.5126	0.0058	<.0001	0.0011	

Supplementary Table S6. Correlation of characteristic lipids in BT-20 cell line (correlation coefficient and p values were listed between every two lipids).

	$C_{16:0}$	$C_{18:0}$	PC(28:0)	PC(30:0)	PC(32:0)	SM(34:0)	$C_{16:1}$	$C_{18:1}$	PC(32:1)	PC(34:1)	PC(36:1)	SM(34:1)	PI(34:1)	PI(36:1)	$C_{20:4}$	$C_{22:4}$	$C_{22:6}$	PC(38:4)	PI(38:4)	PE(36:4)	PE(38:4)	PE(P-38:5)	PE(O-38:5)
$C_{16:0}$	1	0.79978	-0.17241	0.15551	0.24494	-0.1426	0.79088	-0.31791	-0.02469	-0.07542	0.29166	0.08165	-0.52303	-0.81891	0.75751	0.72013	-0.1822	0.01179	-0.25473	-0.64672	0.34194	-0.69344	-0.13993
$C_{16:0}$		<.0001	0.3623	0.4119	0.192	0.4522	<.0001	0.0869	0.8969	0.692	0.1179	0.668	0.003	<.0001	<.0001	<.0001	0.3352	0.9507	0.1743	0.0001	0.0644	<.0001	0.4608
$C_{18:0}$	0.79978	1	-0.2485	0.0812	0.15462	-0.26674	0.6218	-0.35039	0.03849	0.05451	0.29077	-0.03493	-0.59377	-0.79399	0.89855	0.83226	-0.22091	0.07987	-0.01357	-0.83404	0.48654	-0.85362	-0.19377
$C_{18:0}$	<.0001		0.1855	0.6697	0.4146	0.1542	0.0002	0.0577	0.84	0.7748	0.119	0.8546	0.0005	<.0001	<.0001	<.0001	0.2407	0.6748	0.9433	<.0001	0.0064	<.0001	0.3049
PC(28:0)	-0.17241	-0.2485	1	0.43982	-0.29655	0.22047	-0.00868	-0.05495	0.44561	0.15684	-0.11813	0.4109	-0.03092	0.18977	-0.24049	-0.24716	-0.12881	-0.78331	-0.02202	0.1426	0.08788	0.10968	0.11769
PC(28:0)	0.3623	0.1855		0.015	0.1115	0.2417	0.9637	0.773	0.0136	0.4078	0.5341	0.0241	0.8711	0.3152	0.2005	0.1879	0.4975	<.0001	0.908	0.4522	0.6443	0.564	0.5357
PC(30:0)	0.15551	0.0812	0.43982	1	0.22492	0.25873	0.05895	-0.29522	0.2089	0.14438	0.36596	0.70768	-0.28721	-0.06029	0.04071	0.12792	-0.14305	-0.73393	0.08921	-0.14171	0.34238	-0.18354	-0.21424
PC(30:0)	0.4119	0.6697	0.015		0.2321	0.1674	0.757	0.1132	0.2679	0.4465	0.0467	<.0001	0.1238	0.7516	0.8309	0.5005	0.4508	<.0001	0.6392	0.4551	0.064	0.3316	0.2556
PC(32:0)	0.24494	0.15462	-0.29655	0.22492	1	0.09143	0.22447	-0.07542	-0.60489	-0.35528	0.55373	0.19911	-0.00156	-0.1564	0.14438	0.08476	0.00245	0.24494	-0.1515	-0.26986	0.04249	-0.31835	-0.03582
PC(32:0)	0.192	0.4146	0.1115	0.2321		0.6308	0.2331	0.692	0.0004	0.054	0.0015	0.2915	0.9935	0.4092	0.4465	0.6561	0.9898	0.192	0.4242	0.1493	0.8236	0.0864	0.851
SM(34:0)	-0.1426	-0.26674	0.22047	0.25873	0.09143	1	-0.18443	0.11858	-0.06785	0.09143	0.21557	0.61112	-0.03181	0.204	-0.22091	-0.25117	-0.14972	-0.19244	0.06474	0.34638	0.05895	0.26318	-0.10923
SM(34:0)	0.4522	0.1542	0.2417	0.1674	0.6308		0.3292	0.5326	0.7216	0.6308	0.2526	0.0003	0.8675	0.2796	0.2407	0.1806	0.4297	0.3083	0.7339	0.0608	0.757	0.16	0.5656
$C_{16:1}$	0.79088	0.6218	-0.00868	0.05895	0.22447	-0.18443	1	-0.3406	0.08298	0.07987	0.2089	0.08966	-0.26452	-0.69833	0.60356	0.46251	0.08076	-0.05406	-0.61691	-0.6356	0.19778	-0.58398	-0.03404
$C_{16:1}$	<.0001	0.0002	0.9637	0.757	0.2331	0.3292		0.0655	0.6629	0.6748	0.2679	0.6375	0.1578	<.0001	0.0004	0.0101	0.6714	0.7766	0.0003	0.0002	0.2948	0.0007	0.8583
$C_{18:1}$	-0.31791	-0.35039	-0.05495	-0.29522	-0.07542	0.11858	-0.3406	1	-0.1426	-0.03092	-0.13192	-0.27964	0.56618	0.46919	-0.45851	-0.47275	0.54349	0.20667	-0.14794	0.40512	-0.64805	0.35306	0.24449
$C_{18:1}$	0.0869	0.0577	0.773	0.1132	0.692	0.5326	0.0655		0.4522	0.8711	0.4871	0.1345	0.0011	0.0089	0.0108	0.0083	0.0019	0.2732	0.4353	0.0264	0.0001	0.0557	0.1929
PC(32:1)	-0.02469	0.03849	0.44561	0.2089	-0.60489	-0.06785	0.08298	-0.1426	1	0.67519	-0.51235	0.02425	-0.17642	0.00067	0.06474	-0.06652	0.02291	-0.53237	-0.06251	0.16796	0.02781	0.23337	-0.15595
PC(32:1)	0.8969	0.84	0.0136	0.2679	0.0004	0.7216	0.6629	0.4522		<.0001	0.0038	0.8988	0.351	0.9972	0.7339	0.7269	0.9043	0.0025	0.7428	0.375	0.884	0.2146	0.4105
PC(34:1)	-0.07542	0.05451	0.15684	0.14438	-0.35528	0.09143	0.07987	-0.03092	0.67519	1	-0.09499	0.09766	-0.03493	0.02514	0.03404	-0.1208	0.25384	-0.15684	-0.12614	0.1604	0.00245	0.26496	-0.19333
PC(34:1)	0.692	0.7748	0.4078	0.4465	0.054	0.6308	0.6748	0.8711	<.0001		0.6175	0.6077	0.8546	0.8951	0.8583	0.5249	0.1759	0.4078	0.5066	0.3972	0.9898	0.1571	0.306
PC(36:1)	0.29166	0.29077	-0.11813	0.36596	0.55373	0.21557	0.2089	-0.13192	-0.51235	-0.09499	1	0.40245	-0.14082	-0.2703	0.19466	0.21468	-0.00245	0.02247	0.03092	-0.4238	0.402	-0.46563	-0.25339
PC(36:1)	0.1179	0.119	0.5341	0.0467	0.0015	0.2526	0.2679	0.4871	0.0038	0.6175		0.0275	0.4579	0.1486	0.3026	0.2546	0.9898	0.9062	0.8711	0.0196	0.0277	0.0095	0.1767
SM(34:1)	0.08165	-0.03493	0.4109	0.70768	0.19911	0.61112	0.08966	-0.27964	0.02425	0.09766	0.40245	1	-0.21379	-0.05717	-0.00912	0.07008	-0.20623	-0.51991	0.01402	-0.03537	0.26585	-0.13148	-0.16752
SM(34:1)	0.668	0.8546	0.0241	<.0001	0.2915	0.0003	0.6375	0.1345	0.8988	0.6077	0.0275		0.2566	0.7641	0.9618	0.7129	0.2742	0.0032	0.9414	0.8528	0.1556	0.4886	0.3762
PI(34:1)	-0.52303	-0.59377	-0.03092	-0.28721	-0.00156	-0.03181	-0.26452	0.56618	-0.17642	-0.03493	-0.14082	-0.21379	1	0.66541	-0.70812	-0.69121	0.61735	0.15773	-0.35128	0.39533	-0.65428	0.4941	0.38287
PI(34:1)	0.003	0.0005	0.8711	0.1238	0.9935	0.8675	0.1578	0.0011	0.351	0.8546	0.4579	0.2566		<.0001	<.0001	<.0001	0.0003	0.4052	0.057	0.0306	<.0001	0.0055	0.0368
PI(36:1)	-0.81891	-0.79399	0.18977	-0.06029	-0.1564	0.204	-0.69833	0.46919	0.00067	0.02514	-0.2703	-0.05717	0.66541	1	-0.91991	-0.84383	0.2921	-0.04694	0.09677	0.68543	-0.51546	0.70634	0.11502
PI(36:1)	<.0001	<.0001	0.3152	0.7516	0.4092	0.2796	<.0001	0.0089	0.9972	0.8951	0.1486	0.7641	<.0001		<.0001	<.0001	0.1173	0.8054	0.6109	<.0001	0.0036	<.0001	0.545
$C_{20:4}$	0.75751	0.89855	-0.24049	0.04071	0.14438	-0.22091	0.60356	-0.45851	0.06474	0.03404	0.19466	-0.00912	-0.70812	-0.91991	1	0.8723	-0.28899	0.07497	0.0158	-0.73971	0.4723	-0.75706	-0.20801
$C_{20:4}$	<.0001	<.0001	0.2005	0.8309	0.4465	0.2407	0.0004	0.0108	0.7339	0.8583	0.3026	0.9618	<.0001	<.0001		<.0001	0.1214	0.6938	0.934	<.0001	0.0084	<.0001	0.27
$C_{22:4}$	0.72013	0.83226	-0.24716	0.12792	0.08476	-0.25117	0.46251	-0.47275	-0.06652	-0.1208	0.21468	0.07008	-0.69121	-0.84383	0.8723	1	-0.45717	0.05762	0.16752	-0.72013	0.57241	-0.78287	-0.04694
$C_{22:4}$	<.0001	<.0001	0.1879	0.5005	0.6561	0.1806	0.0101	0.0083	0.7269	0.5249	0.2546	0.7129	<.0001	<.0001	<.0001		0.0111	0.7623	0.3762	<.0001	0.0009	<.0001	0.8054
$C_{22:6}$	-0.1822	-0.22091	-0.12881	-0.14305	0.00245	-0.14972	0.08076	0.54349	0.02291	0.25384	-0.00245	-0.20623	0.61735	0.2921	-0.28899	-0.45717	1	0.13192	-0.60445	0.15951	-0.69166	0.27075	-0.03626
$C_{22:6}$	0.3352	0.2407	0.4975	0.4508	0.9898	0.4297	0.6714	0.0019	0.9043	0.1759	0.9898	0.2742	0.0003	0.1173	0.1214	0.0111		0.4871	0.0004	0.3998	<.0001	0.1479	0.8491
PC(38:4)	0.01179	0.07987	-0.78331	-0.73393	0.24494	-0.19244	-0.05406	0.20667	-0.53237	-0.15684	0.02247	-0.51991	0.15773	-0.04694	0.07497	0.05762	0.13192	1	-0.02781	-0.00378	-0.17642	0.02336	0.15506
PC(38:4)	0.9507	0.6748	<.0001	<.0001	0.192	0.3083	0.7766	0.2732	0.0025	0.4078	0.9062	0.0032	0.4052	0.8054	0.6938	0.7623	0.4871		0.884	0.9842	0.351	0.9025	0.4133
PI(38:4)	-0.25473	-0.01357	-0.02202	0.08921	-0.1515	0.06474	-0.61691	-0.14794	-0.06251	-0.12614	0.03092	0.01402	-0.35128	0.09677	0.0158	0.16752	-0.60445	-0.02781	1	0.13593	0.39711	0.0465	-0.11947
PI(38:4)	0.1743	0.9433	0.908	0.6392	0.4242	0.7339	0.0003	0.4353	0.7428	0.5066	0.8711	0.9414	0.057	0.6109	0.934	0.3762	0.0004	0.884		0.4739	0.0298	0.8072	0.5295
PE(36:4)	-0.64672	-0.83404	0.1426	-0.14171	-0.26986	0.34638	-0.6356	0.40512	0.16796	0.1604	-0.4238	-0.03537	0.39533	0.68543	-0.73971	-0.72013	0.15951	-0.00378	0.13593	1	-0.54483	0.9337	-0.03537
PE(36:4)	0.0001	<.0001	0.4522	0.4551	0.1493	0.0608	0.0002	0.0264	0.375	0.3972	0.0196	0.8528	0.0306	<.0001	<.0001	<.0001	0.3998	0.9842	0.4739		0.0019	<.0001	0.8528
PE(38:4)	0.34194	0.48654	0.08788	0.34238	0.04249	0.05895	0.19778	-0.64805	0.02781	0.00245	0.402	0.26585	-0.65428	-0.51546	0.4723	0.57241	-0.69166	-0.17642	0.39711	-0.54483	1	-0.52481	-0.07008
PE(38:4)	0.0644	0.0064	0.6443	0.064	0.8236	0.757	0.2948	0.0001	0.884	0.9898	0.0277	0.1556	<.0001	0.0036	0.0084	0.0009	<.0001	0.351	0.0298	0.0019		0.0029	0.7129
PE(P-38:5	-0.69344	-0.85362	0.10968	-0.18354	-0.31835	0.26318	-0.58398	0.35306	0.23337	0.26496	-0.46563	-0.13148	0.4941	0.70634	-0.75706	-0.78287	0.27075	0.02336	0.0465	0.9337	-0.52481	1	0.02647
PE(P-38:5	<.0001	<.0001	0.564	0.3316	0.0864	0.16	0.0007	0.0557	0.2146	0.1571	0.0095	0.4886	0.0055	<.0001	<.0001	<.0001	0.1479	0.9025	0.8072	<.0001	0.0029		0.8896
PE(O-38:5	-0.13993	-0.19377	0.11769	-0.21424	-0.03582	-0.10923	-0.03404	0.24449	-0.15595	-0.19333	-0.25339	-0.16752	0.38287	0.11502	-0.20801	-0.04694	-0.03626	0.15506	-0.11947	-0.03537	-0.07008	0.02647	1
PE(O-38:5	0.4608	0.3049	0.5357	0.2556	0.851	0.5656	0.8583	0.1929	0.4105	0.306	0.1767	0.3762	0.0368	0.545	0.27	0.8054	0.8491	0.4133	0.5295	0.8528	0.7129	0.8896	

Supplementary Table S7. Correlation of characteristic lipids in MCF-7 cell line (correlation coefficient and *p* values were listed between every two lipids).

	$C_{16:0}$	$C_{18:0}$	PC(28:0)	PC(30:0)	PC(32:0)	SM(34:0)	$C_{16:1}$	$C_{18:1}$	PC(32:1)	PC(34:1)	PC(36:1)	SM(34:1)	PI(34:1)	PI(36:1)	$C_{20:4}$	$C_{22:4}$	$C_{22:6}$	PC(38:4)	PI(38:4)	PE(36:4)	PE(38:4)	PE(P-38:5)	PE(O-38:5)
$C_{16:0}$	1	0.68365	0.33348	0.37086	-0.02024	-0.03938	0.48343	0.61646	0.23337	-0.27653	-0.28409	0.10211	-0.43226	-0.8594	0.58265	0.1782	0.63115	-0.23026	-0.63871	-0.07853	0.13682	-0.01357	-0.27075
$C_{16:0}$		<.0001	0.0717	0.0436	0.9154	0.8363	0.0068	0.0003	0.2146	0.1391	0.1281	0.5913	0.0171	<.0001	0.0007	0.3461	0.0002	0.2209	0.0001	0.68	0.4709	0.9433	0.1479
$C_{18:0}$	0.68365	1	0.41802	0.28053	0.09989	-0.19511	0.05006	0.61913	0.3931	0.0594	-0.25295	0.0683	-0.81179	-0.67742	0.92392	0.70234	0.79533	-0.35662	-0.11279	-0.45139	-0.28543	-0.65117	-0.78198
$C_{18:0}$	<.0001		0.0215	0.1332	0.5995	0.3015	0.7928	0.0003	0.0316	0.7552	0.1775	0.7199	<.0001	<.0001	<.0001	<.0001	<.0001	0.0531	0.5529	0.0123	0.1263	<.0001	<.0001
PC(28:0)	0.33348	0.41802	1	0.73259	-0.22002	-0.41001	0.04739	0.13993	0.63026	-0.05362	-0.28276	-0.38465	-0.46162	-0.2614	0.52436	0.3931	0.39533	-0.42425	0.15951	-0.37219	-0.13281	-0.34994	-0.4376
PC(28:0)	0.0717	0.0215		<.0001	0.2427	0.0244	0.8036	0.4608	0.0002	0.7784	0.13	0.0358	0.0102	0.1629	0.0029	0.0316	0.0306	0.0195	0.3998	0.0428	0.4841	0.058	0.0156
PC(30:0)	0.37086	0.28053	0.73259	1	-0.14394	-0.13103	0.22581	0.14616	0.52659	-0.41935	-0.10478	-0.28365	-0.10256	-0.21913	0.2614	0.12436	0.4287	-0.17464	-0.08432	-0.45539	-0.08209	-0.12747	-0.31657
PC(30:0)	0.0436	0.1332	<.0001		0.4479	0.4901	0.2302	0.4409	0.0028	0.0211	0.5816	0.1288	0.5897	0.2446	0.1629	0.5126	0.0181	0.356	0.6578	0.0114	0.6663	0.502	0.0883
PC(32:0)	-0.02024	0.09989	-0.22002	-0.14394	1	-0.02825	-0.06741	0.06919	-0.36997	0.34816	-0.25562	0.7366	-0.03893	-0.01891	-0.05495	0.13014	-0.12481	-0.15061	0.19822	-0.19288	-0.32369	-0.22047	-0.14616
PC(32:0)	0.9154	0.5995	0.2427	0.4479		0.8822	0.7234	0.7164	0.0442	0.0594	0.1728	<.0001	0.8382	0.921	0.773	0.493	0.5111	0.4269	0.2937	0.3072	0.081	0.2417	0.4409
SM(34:0)	-0.03938	-0.19511	-0.41001	-0.13103	-0.02825	1	-0.0238	-0.15551	-0.57019	-0.57419	0.50478	0.3139	0.1604	-0.03938	-0.28276	-0.13949	-0.18932	0.59155	-0.12347	0.20712	0.15373	0.17197	0.34372
SM(34:0)	0.8363	0.3015	0.0244	0.4901	0.8822		0.9006	0.4119	0.001	0.0009	0.0044	0.0912	0.3972	0.8363	0.13	0.4622	0.3163	0.0006	0.5157	0.2721	0.4173	0.3635	0.0629
$C_{16:1}$	0.48343	0.05006	0.04739	0.22581	-0.06741	-0.0238	1	0.45984	0.00156	-0.23826	-0.25873	-0.00245	0.27253	-0.4069	-0.0238	-0.42202	0.2218	0.02692	-0.60089	0.06785	0.0198	0.40378	0.10834
$C_{16:1}$	0.0068	0.7928	0.8036	0.2302	0.7234	0.9006		0.0106	0.9935	0.2048	0.1674	0.9898	0.1451	0.0256	0.9006	0.0202	0.2388	0.8877	0.0004	0.7216	0.9173	0.0269	0.5688
$C_{18:1}$	0.61646	0.61913	0.13993	0.14616	0.06919	-0.15551	0.45984	1	0.17375	0.03582	-0.11413	-0.07764	-0.38154	-0.61335	0.56841	0.22358	0.58532	-0.05628	-0.46073	-0.34505	-0.27742	-0.1871	-0.46207
$C_{18:1}$	0.0003	0.0003	0.4608	0.4409	0.7164	0.4119	0.0106		0.3585	0.851	0.5482	0.6834	0.0375	0.0003	0.001	0.235	0.0007	0.7677	0.0104	0.0618	0.1377	0.3222	0.0102
PC(32:1)	0.23337	0.3931	0.63026	0.52659	-0.36997	-0.57019	0.00156	0.17375	1	0.19021	-0.29922	-0.56574	-0.27608	-0.1386	0.44472	0.22002	0.5693	-0.53014	-0.03893	-0.41135	-0.17464	-0.30011	-0.47987
PC(32:1)	0.2146	0.0316	0.0002	0.0028	0.0442	0.001	0.9935	0.3585		0.314	0.1082	0.0011	0.1397	0.4651	0.0138	0.2427	0.001	0.0026	0.8382	0.0239	0.356	0.1071	0.0073
PC(34:1)	-0.27653	0.0594	-0.05362	-0.41935	0.34816	-0.57419	-0.23826	0.03582	0.19021	1	-0.48476	0.08966	-0.22581	0.17597	0.10923	0.24761	-0.11235	-0.55284	0.41135	-0.20178	-0.44561	-0.37175	-0.27075
PC(34:1)	0.1391	0.7552	0.7784	0.0211	0.0594	0.0009	0.2048	0.851	0.314		0.0066	0.6375	0.2302	0.3523	0.5656	0.1871	0.5545	0.0015	0.0239	0.2849	0.0136	0.0431	0.1479
PC(36:1)	-0.28409	-0.25295	-0.28276	-0.10478	-0.25562	0.50478	-0.25873	-0.11413	-0.29922	-0.48476	1	-0.16085	0.18576	0.25028	-0.20756	-0.06652	-0.18087	0.71924	-0.05984	0.18977	0.30456	0.10656	0.20934
PC(36:1)	0.1281	0.1775	0.13	0.5816	0.1728	0.0044	0.1674	0.5482	0.1082	0.0066		0.3958	0.3257	0.1822	0.2711	0.7269	0.3388	<.0001	0.7534	0.3152	0.1018	0.5752	0.2669
SM(34:1)	0.10211	0.0683	-0.38465	-0.28365	0.7366	0.3139	-0.00245	-0.07764	-0.56574	0.08966	-0.16085	1	-0.03137	-0.19422	-0.05317	0.01046	-0.12125	-0.11546	-0.02425	0.12836	-0.07675	-0.0723	0.07497
SM(34:1)	0.5913	0.7199	0.0358	0.1288	<.0001	0.0912	0.9898	0.6834	0.0011	0.6375	0.3958		0.8693	0.3038	0.7802	0.9563	0.5233	0.5435	0.8988	0.499	0.6869	0.7042	0.6938
PI(34:1)	-0.43226	-0.81179	-0.46162	-0.10256	-0.03893	0.1604	0.27253	-0.38154	-0.27608	-0.22581	0.18576	-0.03137	1	0.52747	-0.87942	-0.86696	-0.4901	0.3713	-0.21246	0.32236	0.24583	0.76107	0.67164
PI(34:1)	0.0171	<.0001	0.0102	0.5897	0.8382	0.3972	0.1451	0.0375	0.1397	0.2302	0.3257	0.8693		0.0027	<.0001	<.0001	0.006	0.0434	0.2597	0.0823	0.1904	<.0001	<.0001
PI(36:1)	-0.8594	-0.67742	-0.2614	-0.21913	-0.01891	-0.03938	-0.4069	-0.61335	-0.1386	0.17597	0.25028	-0.19422	0.52747	1	-0.57642	-0.18398	-0.59199	0.22447	0.55417	0.10523	-0.01713	0.12347	0.20623
PI(36:1)	<.0001	<.0001	0.1629	0.2446	0.921	0.8363	0.0256	0.0003	0.4651	0.3523	0.1822	0.3038	0.0027		0.0009	0.3304	0.0006	0.2331	0.0015	0.58	0.9284	0.5157	0.2742
$C_{20:4}$	0.58265	0.92392	0.52436	0.2614	-0.05495	-0.28276	-0.0238	0.56841	0.44472	0.10923	-0.20756	-0.05317	-0.87942	-0.57642	1	0.7842	0.73749	-0.40556	-0.00334	-0.39755	-0.25028	-0.69433	-0.77887
$C_{20:4}$	0.0007	<.0001	0.0029	0.1629	0.773	0.13	0.9006	0.001	0.0138	0.5656	0.2711	0.7802	<.0001	0.0009		<.0001	<.0001	0.0262	0.986	0.0296	0.1822	<.0001	<.0001
$C_{22:4}$	0.1782	0.70234	0.3931	0.12436	0.13014	-0.13949	-0.42202	0.22358	0.22002	0.24761	-0.06652	0.01046	-0.86696	-0.18398	0.7842	1	0.40512	-0.25829	0.4772	-0.45451	-0.39132	-0.85317	-0.77486
$C_{22:4}$	0.3461	<.0001	0.0316	0.5126	0.493	0.4622	0.0202	0.235	0.2427	0.1871	0.7269	0.9563	<.0001	0.3304	<.0001		0.0264	0.1682	0.0077	0.0116	0.0325	<.0001	<.0001
$C_{22:6}$	0.63115	0.79533	0.39533	0.4287	-0.12481	-0.18932	0.2218	0.58532	0.5693	-0.11235	-0.18087	-0.12125	-0.4901	-0.59199	0.73749	0.40512	1	-0.31123	-0.33526	-0.41313	-0.18265	-0.44205	-0.67119
$C_{22:6}$	0.0002	<.0001	0.0306	0.0181	0.5111	0.3163	0.2388	0.0007	0.001	0.5545	0.3388	0.5233	0.006	0.0006	<.0001	0.0264		0.0941	0.0701	0.0233	0.334	0.0145	<.0001
PC(38:4)	-0.23026	-0.35662	-0.42425	-0.17464	-0.15061	0.59155	0.02692	-0.05628	-0.53014	-0.55284	0.71924	-0.11546	0.3713	0.22447	-0.40556	-0.25829	-0.31123	1	-0.10923	0.37664	0.40823	0.36997	0.39933
PC(38:4)	0.2209	0.0531	0.0195	0.356	0.4269	0.0006	0.8877	0.7677	0.0026	0.0015	<.0001	0.5435	0.0434	0.2331	0.0262	0.1682	0.0941		0.5656	0.0402	0.0251	0.0442	0.0288
PI(38:4)	-0.63871	-0.11279	0.15951	-0.08432	0.19822	-0.12347	-0.60089	-0.46073	-0.03893	0.41135	-0.05984	-0.02425	-0.21246	0.55417	-0.00334	0.4772	-0.33526	-0.10923	1	-0.27564	-0.40601	-0.52703	-0.28454
PI(38:4)	0.0001	0.5529	0.3998	0.6578	0.2937	0.5157	0.0004	0.0104	0.8382	0.0239	0.7534	0.8988	0.2597	0.0015	0.986	0.0077	0.0701	0.5656		0.1404	0.026	0.0028	0.1275
PE(36:4)	-0.07853	-0.45139	-0.37219	-0.45539	-0.19288	0.20712	0.06785	-0.34505	-0.41135	-0.20178	0.18977	0.12836	0.32236	0.10523	-0.39755	-0.45451	-0.41313	0.37664	-0.27564	1	0.74416	0.596	0.69032
PE(36:4)	0.68	0.0123	0.0428	0.0114	0.3072	0.2721	0.7216	0.0618	0.0239	0.2849	0.3152	0.499	0.0823	0.58	0.0296	0.0116	0.0233	0.0402	0.1404		<.0001	0.0005	<.0001
PE(38:4)	0.13682	-0.28543	-0.13281	-0.08209	-0.32369	0.15373	0.0198	-0.27742	-0.17464	-0.44561	0.30456	-0.07675	0.24583	-0.01713	-0.25028	-0.39132	-0.18265	0.40823	-0.40601	0.74416	1	0.60267	0.60578
PE(38:4)	0.4709	0.1263	0.4841	0.6663	0.081	0.4173	0.9173	0.1377	0.356	0.0136	0.1018	0.6869	0.1904	0.9284	0.1822	0.0325	0.334	0.0251	0.026	<.0001		0.0004	0.0004
PE(P-38:5)	-0.01357	-0.65117	-0.34994	-0.12747	-0.22047	0.17197	0.40378	-0.1871	-0.30011	-0.37175	0.10656	-0.0723	0.76107	0.12347	-0.69433	-0.85317	-0.44205	0.36997	-0.52703	0.596	0.60267	1	0.82603
PE(P-38:5)	0.9433	<.0001	0.058	0.502	0.2417	0.3635	0.0269	0.3222	0.1071	0.0431	0.5752	0.7042	<.0001	0.5157	<.0001	<.0001	0.0145	0.0442	0.0028	0.0005	0.0004		<.0001
PE(O-38:5)	-0.27075	-0.78198	-0.4376	-0.31657	-0.14616	0.34372	0.10834	-0.46207	-0.47987	-0.27075	0.20934	0.07497	0.67164	0.20623	-0.77887	-0.77486	-0.67119	0.39933	-0.28454	0.69032	0.60578	0.82603	1
PE(O-38:5)	0.1479	<.0001	0.0156	0.0883	0.4409	0.0629	0.5688	0.0102	0.0073	0.1479	0.2669	0.6938	<.0001	0.2742	<.0001	<.0001	<.0001	0.0288	0.1275	<.0001	0.0004	<.0001	

Supplementary Table S8. Correlation of characteristic lipids in SK-BR-3 cell line (correlation coefficient and *p* values were listed between every two lipids

	$C_{16:0}$	$C_{18:0}$	PC(28:0)	PC(30:0)	PC(32:0)	SM(34:0)	$C_{16:1}$	$C_{18:1}$	PC(32:1)	PC(34:1)	PC(36:1)	SM(34:1)	PI(34:1)	PI(36:1)	$C_{20:4}$	$C_{22:4}$	$C_{22:6}$	PC(38:4)	PI(38:4)	PE(36:4)	PE(38:4)	PE(P-38:5)	PE(O-38:5)
$C_{16:0}$	1	0.83003	0.12436	0.23871	0.26808	-0.09143	0.60133	0.78509	0.1564	0.09277	0.14705	0.0594	-0.62937	-0.52214	0.42336	0.19422	0.79088	-0.26496	-0.44828	-0.05095	0.28098	-0.27386	-0.02158
$C_{16:0}$		<.0001	0.5126	0.204	0.1521	0.6308	0.0004	<.0001	0.4092	0.6258	0.4381	0.7552	0.0002	0.0031	0.0197	0.3038	<.0001	0.1571	0.013	0.7892	0.1326	0.1431	0.9099
$C_{18:0}$	0.83003	1	0.02825	0.28854	0.28231	-0.23159	0.51012	0.83582	0.10612	0.17375	0.22403	0.07186	-0.57775	-0.44828	0.55551	0.19288	0.80601	-0.18621	-0.22136	-0.08966	0.06162	-0.29967	-0.15907
$C_{18:0}$	<.0001		0.8822	0.122	0.1307	0.2182	0.004	<.0001	0.5768	0.3585	0.234	0.7059	0.0008	0.013	0.0014	0.3072	<.0001	0.3245	0.2398	0.6375	0.7463	0.1077	0.4011
PC(28:0)	0.12436	0.02825	1	0.29655	-0.30945	-0.33259	0.30723	0.13059	0.27119	-0.51947	-0.0238	-0.36285	0.05362	0.13637	-0.0287	0.43092	0.17775	-0.35083	-0.1297	-0.12792	-0.2921	-0.32325	-0.30456
PC(28:0)	0.5126	0.8822		0.1115	0.0961	0.0725	0.0986	0.4916	0.1472	0.0033	0.9006	0.0488	0.7784	0.4724	0.8803	0.0174	0.3474	0.0573	0.4945	0.5005	0.1173	0.0814	0.1018
PC(30:0)	0.23871	0.28854	0.29655	1	0.32414	-0.18042	0.56352	0.39844	0.34149	0.23826	0.13237	0.31969	0.11902	0.24627	-0.15417	0.19867	0.32414	-0.17286	-0.42425	-0.2881	-0.3931	-0.47408	-0.47275
PC(30:0)	0.204	0.122	0.1115		0.0806	0.34	0.0012	0.0292	0.0648	0.2048	0.4856	0.085	0.531	0.1896	0.416	0.2926	0.0806	0.361	0.0195	0.1226	0.0316	0.0081	0.0083
PC(32:0)	0.26808	0.28231	-0.30945	0.32414	1	0.02736	0.16396	0.1644	-0.24182	0.25962	-0.25206	0.44917	-0.32636	-0.22358	0.10256	-0.27697	0.11947	-0.14171	-0.22225	0.22091	0.26941	-0.03315	0.19867
PC(32:0)	0.1521	0.1307	0.0961	0.0806		0.8859	0.3866	0.3853	0.1979	0.1659	0.179	0.0128	0.0784	0.235	0.5897	0.1384	0.5295	0.4551	0.2378	0.2407	0.15	0.862	0.2926
SM(34:0)	-0.09143	-0.23159	-0.33259	-0.18042	0.02736	1	-0.02558	-0.14661	0.08832	0.30901	-0.27208	0.46251	-0.06563	-0.01135	-0.21068	-0.29967	-0.02336	-0.06029	-0.20756	-0.01846	0.07364	0.23826	0.28721
SM(34:0)	0.6308	0.2182	0.0725	0.34	0.8859		0.8932	0.4395	0.6426	0.0966	0.1458	0.0101	0.7304	0.9525	0.2638	0.1077	0.9025	0.7516	0.2711	0.9228	0.699	0.2048	0.1238
$C_{16:1}$	0.60133	0.51012	0.30723	0.56352	0.16396	-0.02558	1	0.80734	0.48476	0.22581	0.18042	0.36418	0.07853	0.2396	-0.3099	0.13993	0.66986	-0.34816	-0.88432	-0.60623	-0.24583	-0.74327	-0.48832
$C_{16:1}$	0.0004	0.004	0.0986	0.0012	0.3866	0.8932		<.0001	0.0066	0.2302	0.34	0.0479	0.68	0.2022	0.0956	0.4608	<.0001	0.0594	<.0001	0.0004	0.1904	<.0001	0.0062
$C_{18:1}$	0.78509	0.83582	0.13059	0.39844	0.1644	-0.14661	0.80734	1	0.27208	0.13815	0.3139	0.13326	-0.25784	-0.12392	0.19155	0.19066	0.87942	-0.29655	-0.56707	-0.47275	-0.12925	-0.59199	-0.43715
$C_{18:1}$	<.0001	<.0001	0.4916	0.0292	0.3853	0.4395	<.0001		0.1458	0.4666	0.0912	0.4827	0.1689	0.5141	0.3106	0.3129	<.0001	0.1115	0.0011	0.0083	0.496	0.0006	0.0157
PC(32:1)	0.1564	0.10612	0.27119	0.34149	-0.24182	0.08832	0.48476	0.27208	1	0.46207	0.16396	0.41535	0.20934	0.23782	-0.33882	0.25295	0.24138	-0.01046	-0.42247	-0.4376	-0.03181	-0.23737	-0.31301
PC(32:1)	0.4092	0.5768	0.1472	0.0648	0.1979	0.6426	0.0066	0.1458		0.0102	0.3866	0.0225	0.2669	0.2057	0.067	0.1775	0.1988	0.9563	0.02	0.0156	0.8675	0.2066	0.0921
PC(34:1)	0.09277	0.17375	-0.51947	0.23826	0.25962	0.30901	0.22581	0.13815	0.46207	1	0.19733	0.73882	0.0287	0.0594	-0.19199	-0.12925	0.07364	0.24805	-0.2921	-0.17775	0.12792	-0.03849	0.09588
PC(34:1)	0.6258	0.3585	0.0033	0.2048	0.1659	0.0966	0.2302	0.4666	0.0102		0.2959	<.0001	0.8803	0.7552	0.3094	0.496	0.699	0.1863	0.1173	0.3474	0.5005	0.84	0.6142
PC(36:1)	0.14705	0.22403	-0.0238	0.13237	-0.25206	-0.27208	0.18042	0.3139	0.16396	0.19733	1	-0.02647	0.19288	0.15551	-0.01535	0.11279	0.18799	0.29255	-0.15195	-0.297	-0.15595	-0.15373	-0.14527
PC(36:1)	0.4381	0.234	0.9006	0.4856	0.179	0.1458	0.34	0.0912	0.3866	0.2959		0.8896	0.3072	0.4119	0.9358	0.5529	0.3198	0.1167	0.4228	0.111	0.4105	0.4173	0.4437
SM(34:1)	0.0594	0.07186	-0.36285	0.31969	0.44917	0.46251	0.36418	0.13326	0.41535	0.73882	-0.02647	1	0.12659	0.23471	-0.40868	-0.02514	0.12747	-0.16841	-0.49811	-0.2089	0.06207	-0.14216	0.03003
SM(34:1)	0.7552	0.7059	0.0488	0.085	0.0128	0.0101	0.0479	0.4827	0.0225	<.0001	0.8896		0.5051	0.2119	0.0249	0.8951	0.502	0.3737	0.0051	0.2679	0.7445	0.4536	0.8748
PI(34:1)	-0.62937	-0.57775	0.05362	0.11902	-0.32636	-0.06563	0.07853	-0.25784	0.20934	0.0287	0.19288	0.12659	1	0.93637	-0.82736	-0.07008	-0.41624	0.11324	-0.18443	-0.54972	-0.42336	-0.35573	-0.48165
PI(34:1)	0.0002	0.0008	0.7784	0.531	0.0784	0.7304	0.68	0.1689	0.2669	0.8803	0.3072	0.5051		<.0001	<.0001	0.7129	0.0221	0.5513	0.3292	0.0017	0.0197	0.0537	0.007
PI(36:1)	-0.52214	-0.44828	0.13637	0.24627	-0.22358	-0.01135	0.2396	-0.12392	0.23782	0.0594	0.15551	0.23471	0.93637	1	-0.83671	0.00467	-0.24405	-0.00289	-0.3139	-0.64405	-0.50701	-0.54616	-0.62581
PI(36:1)	0.0031	0.013	0.4724	0.1896	0.235	0.9525	0.2022	0.5141	0.2057	0.7552	0.4119	0.2119	<.0001		<.0001	0.9805	0.1937	0.9879	0.0912	0.0001	0.0042	0.0018	0.0002
$C_{20:4}$	0.42336	0.55551	-0.0287	-0.15417	0.10256	-0.21068	-0.3099	0.19155	-0.33882	-0.19199	-0.01535	-0.40868	-0.82736	-0.83671	1	0.09232	0.32725	-0.0198	0.54305	0.57197	0.19021	0.42202	0.33971
$C_{20:4}$	0.0197	0.0014	0.8803	0.416	0.5897	0.2638	0.0956	0.3106	0.067	0.3094	0.9358	0.0249	<.0001	<.0001		0.6275	0.0775	0.9173	0.0019	0.001	0.314	0.0202	0.0663
$C_{22:4}$	0.19422	0.19288	0.43092	0.19867	-0.27697	-0.29967	0.13993	0.19066	0.25295	-0.12925	0.11279	-0.02514	-0.07008	0.00467	0.09232	1	0.21602	-0.15462	-0.04338	-0.18665	-0.08699	-0.31346	-0.35929
$C_{22:4}$	0.3038	0.3072	0.0174	0.2926	0.1384	0.1077	0.4608	0.3129	0.1775	0.496	0.5529	0.8951	0.7129	0.9805	0.6275		0.2516	0.4146	0.8199	0.3233	0.6476	0.0917	0.0512
$C_{22:6}$	0.79088	0.80601	0.17775	0.32414	0.11947	-0.02336	0.66986	0.87942	0.24138	0.07364	0.18799	0.12747	-0.41624	-0.24405	0.32725	0.21602	1	-0.37219	-0.45006	-0.28187	-0.07186	-0.42336	-0.33081
$C_{22:6}$	<.0001	<.0001	0.3474	0.0806	0.5295	0.9025	<.0001	<.0001	0.1988	0.699	0.3198	0.502	0.0221	0.1937	0.0775	0.2516		0.0428	0.0126	0.1313	0.7059	0.0197	0.0742
PC(38:4)	-0.26496	-0.18621	-0.35083	-0.17286	-0.14171	-0.06029	-0.34816	-0.29655	-0.01046	0.24805	0.29255	-0.16841	0.11324	-0.00289	-0.0198	-0.15462	-0.37219	1	0.22892	-0.02514	0.16796	0.27519	0.03938
PC(38:4)	0.1571	0.3245	0.0573	0.361	0.4551	0.7516	0.0594	0.1115	0.9563	0.1863	0.1167	0.3737	0.5513	0.9879	0.9173	0.4146	0.0428		0.2237	0.8951	0.375	0.1411	0.8363
PI(38:4)	-0.44828	-0.22136	-0.1297	-0.42425	-0.22225	-0.20756	-0.88432	-0.56707	-0.42247	-0.2921	-0.15195	-0.49811	-0.18443	-0.3139	0.54305	-0.04338	-0.45006	0.22892	1	0.56129	0.13192	0.60534	0.30857
PI(38:4)	0.013	0.2398	0.4945	0.0195	0.2378	0.2711	<.0001	0.0011	0.02	0.1173	0.4228	0.0051	0.3292	0.0912	0.0019	0.8199	0.0126	0.2237		0.0013	0.4871	0.0004	0.0971
PE(36:4)	-0.05095	-0.08966	-0.12792	-0.2881	0.22091	-0.01846	-0.60623	-0.47275	-0.4376	-0.17775	-0.297	-0.2089	-0.54972	-0.64405	0.57197	-0.18665	-0.28187	-0.02514	0.56129	1	0.26941	0.75484	0.7584
PE(36:4)	0.7892	0.6375	0.5005	0.1226	0.2407	0.9228	0.0004	0.0083	0.0156	0.3474	0.111	0.2679	0.0017	0.0001	0.001	0.3233	0.1313	0.8951	0.0013		0.15	<.0001	<.0001
PE(38:4)	0.28098	0.06162	-0.2921	-0.3931	0.26941	0.07364	-0.24583	-0.12925	-0.03181	0.12792	-0.15595	0.06207	-0.42336	-0.50701	0.19021	-0.08699	-0.07186	0.16796	0.13192	0.26941	1	0.35751	0.44739
PE(38:4)	0.1326	0.7463	0.1173	0.0316	0.15	0.699	0.1904	0.496	0.8675	0.5005	0.4105	0.7445	0.0197	0.0042	0.314	0.6476	0.7059	0.375	0.4871	0.15		0.0524	0.0132
PE(P-38:5)	-0.27386	-0.29967	-0.32325	-0.47408	-0.03315	0.23826	-0.74327	-0.59199	-0.23737	-0.03849	-0.15373	-0.14216	-0.35573	-0.54616	0.42202	-0.31346	-0.42336	0.27519	0.60534	0.75484	0.35751	1	0.78287
PE(P-38:5)	0.1431	0.1077	0.0814	0.0081	0.862	0.2048	<.0001	0.0006	0.2066	0.84	0.4173	0.4536	0.0537	0.0018	0.0202	0.0917	0.0197	0.1411	0.0004	<.0001	0.0524		<.0001
PE(O-38:5)	-0.02158	-0.15907	-0.30456	-0.47275	0.19867	0.28721	-0.48832	-0.43715	-0.31301	0.09588	-0.14527	0.03003	-0.48165	-0.62581	0.33971	-0.35929	-0.33081	0.03938	0.30857	0.7584	0.44739	0.78287	1
PE(O-38:5)	0.9099	0.4011	0.1018	0.0083	0.2926	0.1238	0.0062	0.0157	0.0921	0.6142	0.4437	0.8748	0.007	0.0002	0.0663	0.0512	0.0742	0.8363	0.0971	<.0001	0.0132	<.0001	

Supplementary Table S9. Correlation of characteristic lipids in MDA-MB-231 cell line (correlation coefficient and *p* values were listed between every two

	$C_{16:0}$	$C_{18:0}$	PC(28:0)	PC(30:0)	PC(32:0)	SM(34:0)	$C_{16:1}$	$C_{18:1}$	PC(32:1)	PC(34:1)	PC(36:1)	SM(34:1)	PI(34:1)	PI(36:1)	$C_{20:4}$	$C_{22:4}$	$C_{22:6}$	PC(38:4)	PI(38:4)	PE(36:4)	PE(38:4)	PE(P-38:5)	PE(O-38:5)
**$C_{16:0}$**	1	0.83982	-0.06251	-0.04383	-0.12481	0.19733	0.798	0.68365	0.10122	0.17597	-0.08254	0.1257	-0.60667	-0.37842	0.71168	-0.18131	0.64004	-0.13682	-0.84427	0.20934	-0.33037	0.35528	0.57375
**$C_{16:0}$**		<.0001	0.7428	0.8181	0.5111	0.2959	<.0001	<.0001	0.5946	0.3523	0.6646	0.5081	0.0004	0.0392	<.0001	0.3376	0.0001	0.4709	<.0001	0.2669	0.0746	0.054	0.0009
**$C_{18:0}$**	0.83982	1	-0.14082	0.04961	-0.04605	0.32814	0.73749	0.65784	0.07764	0.11991	-0.11902	0.15462	-0.59956	-0.18799	0.76196	0.02425	0.35706	-0.06207	-0.69344	-0.10612	-0.28187	0.02692	0.30189
**$C_{18:0}$**	<.0001		0.4579	0.7946	0.8091	0.0767	<.0001	<.0001	0.6834	0.5279	0.531	0.4146	0.0005	0.3198	<.0001	0.8988	0.0527	0.7445	<.0001	0.5768	0.1313	0.8877	0.1049
**PC(28:0)**	-0.06251	-0.14082	1	-0.1386	-0.15729	-0.39221	-0.11591	0.00556	0.36863	0.11769	0.10167	-0.28587	-0.03982	-0.18087	-0.01268	-0.05984	0.20934	-0.14127	0.16707	0.05584	0.06073	0.00823	0.28009
**PC(28:0)**	0.7428	0.4579		0.4651	0.4065	0.0321	0.5419	0.9767	0.045	0.5357	0.5929	0.1257	0.8345	0.3388	0.947	0.7534	0.2669	0.4565	0.3775	0.7695	0.7499	0.9656	0.1338
**PC(30:0)**	-0.04383	0.04961	-0.1386	1	0.10256	0.08921	0.06518	-0.01402	-0.10834	0.03982	-0.07675	0.33126	-0.0723	0.02514	0.03938	-0.0941	-0.07809	-0.002	0.01357	0.01802	0.15061	-0.08966	0.22625
**PC(30:0)**	0.8181	0.7946	0.4651		0.5897	0.6392	0.7322	0.9414	0.5688	0.8345	0.6869	0.0738	0.7042	0.8951	0.8363	0.6209	0.6817	0.9916	0.9433	0.9247	0.4269	0.6375	0.2293
**PC(32:0)**	-0.12481	-0.04605	-0.15729	0.10256	1	0.04472	-0.03181	0.02336	-0.37753	-0.76774	-0.65295	0.46919	-0.04116	0.20801	-0.23471	0.03626	-0.38376	-0.69032	0.04917	-0.26897	-0.01802	-0.13014	-0.05139
**PC(32:0)**	0.5111	0.8091	0.4065	0.5897		0.8145	0.8675	0.9025	0.0397	<.0001	<.0001	0.0089	0.829	0.27	0.2119	0.8491	0.0363	<.0001	0.7964	0.1507	0.9247	0.493	0.7874
**SM(34:0)**	0.19733	0.32814	-0.39221	0.08921	0.04472	1	0.24894	0.30945	0.04872	0.22581	0.06296	0.28543	0.07408	0.06696	0.05806	0.14794	0.02425	0.03315	-0.28009	-0.47853	-0.36062	-0.12525	-0.1693
**SM(34:0)**	0.2959	0.0767	0.0321	0.6392	0.8145		0.1846	0.0961	0.7982	0.2302	0.741	0.1263	0.6972	0.7252	0.7605	0.4353	0.8988	0.862	0.1338	0.0075	0.0503	0.5096	0.3711
**$C_{16:1}$**	0.798	0.73749	-0.11591	0.06518	-0.03181	0.24894	1	0.79666	0.20934	0.09366	-0.32369	0.02603	-0.40823	-0.05273	0.49277	0.12481	0.31479	-0.07364	-0.90923	0.10745	-0.25873	0.15417	0.39978
**$C_{16:1}$**	<.0001	<.0001	0.5419	0.7322	0.8675	0.1846		<.0001	0.2669	0.6225	0.081	0.8914	0.0251	0.782	0.0057	0.5111	0.0902	0.699	<.0001	0.572	0.1674	0.416	0.0286
**$C_{18:1}$**	0.68365	0.65784	0.00556	-0.01402	0.02336	0.30945	0.79666	1	0.24138	0.04205	-0.22803	-0.01046	-0.31168	0.00556	0.41046	0.15862	0.16352	-0.12659	-0.83671	-0.17063	-0.49143	-0.13014	0.27519
**$C_{18:1}$**	<.0001	<.0001	0.9767	0.9414	0.9025	0.0961	<.0001		0.1988	0.8254	0.2255	0.9563	0.0936	0.9767	0.0243	0.4025	0.3879	0.5051	<.0001	0.3673	0.0058	0.493	0.1411
**PC(32:1)**	0.10122	0.07764	0.36863	-0.10834	-0.37753	0.04872	0.20934	0.24138	1	0.59021	-0.00512	-0.64538	-0.15373	-0.06073	0.13593	0.18487	0.18309	-0.03537	-0.10256	0.0901	-0.13548	-0.103	0.17019
**PC(32:1)**	0.5946	0.6834	0.045	0.5688	0.0397	0.7982	0.2669	0.1988		0.0006	0.9786	0.0001	0.4173	0.7499	0.4739	0.3281	0.3328	0.8528	0.5897	0.6359	0.4753	0.5881	0.3686
**PC(34:1)**	0.17597	0.11991	0.11769	0.03982	-0.76774	0.22581	0.09366	0.04205	0.59021	1	0.52436	-0.38776	-0.03092	-0.0812	0.22403	-0.2218	0.301	0.47186	-0.15729	0.21691	-0.02558	0.13815	0.09855
**PC(34:1)**	0.3523	0.5279	0.5357	0.8345	<.0001	0.2302	0.6225	0.8254	0.0006		0.0029	0.0342	0.8711	0.6697	0.234	0.2388	0.106	0.0085	0.4065	0.2496	0.8932	0.4666	0.6044
**PC(36:1)**	-0.08254	-0.11902	0.10167	-0.07675	-0.65295	0.06296	-0.32369	-0.22803	-0.00512	0.52436	1	-0.01313	0.23337	-0.09855	-0.10345	-0.3139	0.10478	0.4812	0.20934	0.02959	-0.10612	0.01802	0.02959
**PC(36:1)**	0.6646	0.531	0.5929	0.6869	<.0001	0.741	0.081	0.2255	0.9786	0.0029		0.9451	0.2146	0.6044	0.5864	0.0912	0.5816	0.0071	0.2669	0.8767	0.5768	0.9247	0.8767
**SM(34:1)**	0.1257	0.15462	-0.28587	0.33126	0.46919	0.28543	0.02603	-0.01046	-0.64538	-0.38776	-0.01313	1	0.03448	0.00823	-0.08654	-0.30545	-0.15284	-0.27386	-0.0901	-0.14527	-0.1911	0.0772	0.07497
**SM(34:1)**	0.5081	0.4146	0.1257	0.0738	0.0089	0.1263	0.8914	0.9563	0.0001	0.0342	0.9451		0.8564	0.9656	0.6493	0.1007	0.4201	0.1431	0.6359	0.4437	0.3117	0.6851	0.6938
**PI(34:1)**	-0.60667	-0.59956	-0.03982	-0.0723	-0.04116	0.07408	-0.40823	-0.31168	-0.15373	-0.03092	0.23337	0.03448	1	0.35573	-0.66318	0.13281	-0.45717	0.13237	0.33971	-0.38154	-0.06474	-0.29967	-0.38865
**PI(34:1)**	0.0004	0.0005	0.8345	0.7042	0.829	0.6972	0.0251	0.0936	0.4173	0.8711	0.2146	0.8564		0.0537	<.0001	0.4841	0.0111	0.4856	0.0663	0.0375	0.7339	0.1077	0.0338
**PI(36:1)**	-0.37842	-0.18799	-0.18087	0.02514	0.20801	0.06696	-0.05273	0.00556	-0.06073	-0.0812	-0.09855	0.00823	0.35573	1	-0.3931	0.20667	-0.69878	0.0861	0.06251	-0.24716	0.08298	-0.42202	-0.37931
**PI(36:1)**	0.0392	0.3198	0.3388	0.8951	0.27	0.7252	0.782	0.9767	0.7499	0.6697	0.6044	0.9656	0.0537		0.0316	0.2732	<.0001	0.651	0.7428	0.1879	0.6629	0.0202	0.0387
**$C_{20:4}$**	0.71168	0.76196	-0.01268	0.03938	-0.23471	0.05806	0.49277	0.41046	0.13593	0.22403	-0.10345	-0.08654	-0.66318	-0.3931	1	0.04427	0.64627	0.13014	-0.49766	0.19333	-0.09588	0.31702	0.38465
**$C_{20:4}$**	<.0001	<.0001	0.947	0.8363	0.2119	0.7605	0.0057	0.0243	0.4739	0.234	0.5864	0.6493	<.0001	0.0316		0.8163	0.0001	0.493	0.0051	0.306	0.6142	0.0878	0.0358
**$C_{22:4}$**	-0.18131	0.02425	-0.05984	-0.0941	0.03626	0.14794	0.12481	0.15862	0.18487	-0.2218	-0.3139	-0.30545	0.13281	0.20667	0.04427	1	-0.12525	0.08921	0.05095	-0.34638	0.05139	-0.4594	-0.37798
**$C_{22:4}$**	0.3376	0.8988	0.7534	0.6209	0.8491	0.4353	0.5111	0.4025	0.3281	0.2388	0.0912	0.1007	0.4841	0.2732	0.8163		0.5096	0.6392	0.7892	0.0608	0.7874	0.0107	0.0395
**$C_{22:6}$**	0.64004	0.35706	0.20934	-0.07809	-0.38376	0.02425	0.31479	0.16352	0.18309	0.301	0.10478	-0.15284	-0.45717	-0.69878	0.64627	-0.12525	1	0.06652	-0.35395	0.4238	-0.03893	0.61379	0.56707
**$C_{22:6}$**	0.0001	0.0527	0.2669	0.6817	0.0363	0.8988	0.0902	0.3879	0.3328	0.106	0.5816	0.4201	0.0111	<.0001	0.0001	0.5096		0.7269	0.055	0.0196	0.8382	0.0003	0.0011
**PC(38:4)**	-0.13682	-0.06207	-0.14127	-0.002	-0.69032	0.03315	-0.07364	-0.12659	-0.03537	0.47186	0.4812	-0.27386	0.13237	0.0861	0.13014	0.08921	0.06652	1	0.03493	0.12481	0.1782	0.01135	-0.26585
**PC(38:4)**	0.4709	0.7445	0.4565	0.9916	<.0001	0.862	0.699	0.5051	0.8528	0.0085	0.0071	0.1431	0.4856	0.651	0.493	0.6392	0.7269		0.8546	0.5111	0.3461	0.9525	0.1556
**PI(38:4)**	-0.84427	-0.69344	0.16707	0.01357	0.04917	-0.28009	-0.90923	-0.83671	-0.10256	-0.15729	0.20934	-0.0901	0.33971	0.06251	-0.49766	0.05095	-0.35395	0.03493	1	-0.12303	0.39043	-0.22225	-0.39266
**PI(38:4)**	<.0001	<.0001	0.3775	0.9433	0.7964	0.1338	<.0001	<.0001	0.5897	0.4065	0.2669	0.6359	0.0663	0.7428	0.0051	0.7892	0.055	0.8546		0.5172	0.0329	0.2378	0.0318
**PE(36:4)**	0.20934	-0.10612	0.05584	0.01802	-0.26897	-0.47853	0.10745	-0.17063	0.0901	0.21691	0.02959	-0.14527	-0.38154	-0.24716	0.19333	-0.34638	0.4238	0.12481	-0.12303	1	0.48654	0.73526	0.43715
**PE(36:4)**	0.2669	0.5768	0.7695	0.9247	0.1507	0.0075	0.572	0.3673	0.6359	0.2496	0.8767	0.4437	0.0375	0.1879	0.306	0.0608	0.0196	0.5111	0.5172		0.0064	<.0001	0.0157
**PE(38:4)**	-0.33037	-0.28187	0.06073	0.15061	-0.01802	-0.36062	-0.25873	-0.49143	-0.13548	-0.02558	-0.10612	-0.1911	-0.06474	0.08298	-0.09588	0.05139	-0.03893	0.1782	0.39043	0.48654	1	0.28142	-0.04071
**PE(38:4)**	0.0746	0.1313	0.7499	0.4269	0.9247	0.0503	0.1674	0.0058	0.4753	0.8932	0.5768	0.3117	0.7339	0.6629	0.6142	0.7874	0.8382	0.3461	0.0329	0.0064		0.1319	0.8309
**PE(P-38:5)**	0.35528	0.02692	0.00823	-0.08966	-0.13014	-0.12525	0.15417	-0.13014	-0.103	0.13815	0.01802	0.0772	-0.29967	-0.42202	0.31702	-0.4594	0.61379	0.01135	-0.22225	0.73526	0.28142	1	0.59911
**PE(P-38:5)**	0.054	0.8877	0.9656	0.6375	0.493	0.5096	0.416	0.493	0.5881	0.4666	0.9247	0.6851	0.1077	0.0202	0.0878	0.0107	0.0003	0.9525	0.2378	<.0001	0.1319		0.0005
**PE(O-38:5)**	0.57375	0.30189	0.28009	0.22625	-0.05139	-0.1693	0.39978	0.27519	0.17019	0.09855	0.02959	0.07497	-0.38865	-0.37931	0.38465	-0.37798	0.56707	-0.26585	-0.39266	0.43715	-0.04071	0.59911	1
**PE(O-38:5)**	0.0009	0.1049	0.1338	0.2293	0.7874	0.3711	0.0286	0.1411	0.3686	0.6044	0.8767	0.6938	0.0338	0.0387	0.0358	0.0395	0.0011	0.1556	0.0318	0.0157	0.8309	0.0005	

Supplementary Table S10. Correlation of characteristic lipids in MDA-MB-157 cell line (correlation coefficient and *p* values were listed between every two

	$C_{16:0}$	$C_{18:0}$	PC(28:0)	PC(30:0)	PC(32:0)	SM(34:0)	$C_{16:1}$	$C_{18:1}$	PC(32:1)	PC(34:1)	PC(36:1)	SM(34:1)	PI(34:1)	PI(36:1)	$C_{20:4}$	$C_{22:4}$	$C_{22:6}$	PC(38:4)	PI(38:4)	PE(36:4)	PE(38:4)	PE(P-38:5)	PE(O-38:5)
**$C_{16:0}$**	1	0.85984	0.204	0.44605	0.27075	0.30412	0.8376	0.57063	0.26674	0.44071	0.30189	0.25873	-0.20178	-0.23293	0.56618	0.28676	0.12303	-0.32191	-0.53993	-0.42158	-0.13993	-0.42558	-0.1475
**$C_{16:0}$**		<.0001	0.2796	0.0135	0.1479	0.1023	<.0001	0.001	0.1542	0.0148	0.1049	0.1674	0.2849	0.2155	0.0011	0.1244	0.5172	0.0828	0.0021	0.0203	0.4608	0.019	0.4367
**$C_{18:0}$**	0.85984	1	0.25028	0.38376	0.24405	0.34149	0.76952	0.57731	0.20756	0.39755	0.20667	0.06652	-0.14527	-0.09588	0.65072	0.32948	-0.05717	-0.29344	-0.43582	-0.42514	-0.26763	-0.59644	-0.22714
**$C_{18:0}$**	<.0001		0.1822	0.0363	0.1937	0.0648	<.0001	0.0008	0.2711	0.0296	0.2732	0.7269	0.4437	0.6142	<.0001	0.0754	0.7641	0.1155	0.0161	0.0192	0.1528	0.0005	0.2274
**PC(28:0)**	0.204	0.25028	1	-0.03715	0.40645	0.74994	0.32102	0.04917	0.32681	0.30768	-0.32547	-0.12925	-0.11502	-0.06073	0.28632	0.10523	0.09811	0.09143	-0.26674	-0.24672	-0.18799	-0.18843	0.03226
**PC(28:0)**	0.2796	0.1822		0.8455	0.0258	<.0001	0.0837	0.7964	0.078	0.0981	0.0792	0.496	0.545	0.7499	0.1251	0.58	0.606	0.6308	0.1542	0.1887	0.3198	0.3187	0.8656
**PC(30:0)**	0.44605	0.38376	-0.03715	1	-0.1297	-0.14527	0.44383	0.0861	0.36107	0.54972	0.32414	0.34994	-0.13949	-0.17019	0.30145	0.27253	0.05451	-0.53904	-0.20712	-0.06118	0.16263	0.0416	0.20356
**PC(30:0)**	0.0135	0.0363	0.8455		0.4945	0.4437	0.014	0.651	0.05	0.0017	0.0806	0.058	0.4622	0.3686	0.1055	0.1451	0.7748	0.0021	0.2721	0.7481	0.3905	0.8272	0.2806
**PC(32:0)**	0.27075	0.24405	0.40645	-0.1297	1	0.25295	0.2832	0.00823	0.01624	0.0683	-0.15328	0.2614	-0.19288	-0.18087	0.19644	0.08209	0.02469	0.41135	-0.05362	-0.29922	-0.18265	-0.16796	-0.01891
**PC(32:0)**	0.1479	0.1937	0.0258	0.4945		0.1775	0.1294	0.9656	0.9321	0.7199	0.4187	0.1629	0.3072	0.3388	0.2981	0.6663	0.8969	0.0239	0.7784	0.1082	0.334	0.375	0.921
**SM(34:0)**	0.30412	0.34149	0.74994	-0.14527	0.25295	1	0.30901	0.23204	0.23337	0.2	-0.36596	-0.08031	0.06296	0.14483	0.17241	-0.05984	0.01758	0.05317	-0.26808	-0.13415	-0.24182	-0.17108	-0.2485
**SM(34:0)**	0.1023	0.0648	<.0001	0.4437	0.1775		0.0966	0.2173	0.2146	0.2893	0.0467	0.6731	0.741	0.4451	0.3623	0.7534	0.9266	0.7802	0.1521	0.4797	0.1979	0.366	0.1855
**$C_{16:1}$**	0.8376	0.76952	0.32102	0.44383	0.2832	0.30901	1	0.50122	0.2921	0.43493	0.15595	0.15684	-0.05717	-0.08165	0.62759	0.23159	0.07987	-0.34727	-0.60133	-0.36062	-0.36997	-0.43715	-0.10834
**$C_{16:1}$**	<.0001	<.0001	0.0837	0.014	0.1294	0.0966		0.0048	0.1173	0.0163	0.4105	0.4078	0.7641	0.668	0.0002	0.2182	0.6748	0.0601	0.0004	0.0503	0.0442	0.0157	0.5688
**$C_{18:1}$**	0.57063	0.57731	0.04917	0.0861	0.00823	0.23204	0.50122	1	-0.05806	-0.0109	0.04338	-0.22981	0.43715	0.40556	0.05762	-0.32369	-0.19867	-0.10567	-0.39844	-0.04027	-0.13771	-0.28988	-0.73882
**$C_{18:1}$**	0.001	0.0008	0.7964	0.651	0.9656	0.2173	0.0048		0.7605	0.9544	0.8199	0.2218	0.0157	0.0262	0.7623	0.081	0.2926	0.5784	0.0292	0.8327	0.468	0.1202	<.0001
**PC(32:1)**	0.26674	0.20756	0.32681	0.36107	0.01624	0.23337	0.2921	-0.05806	1	0.83226	0.15417	0.08031	-0.11635	-0.04605	0.07898	0.16307	0.13326	-0.33749	-0.13815	-0.02514	0.02558	-0.10567	0.06296
**PC(32:1)**	0.1542	0.2711	0.078	0.05	0.9321	0.2146	0.1173	0.7605		<.0001	0.416	0.6731	0.5403	0.8091	0.6783	0.3892	0.4827	0.0682	0.4666	0.8951	0.8932	0.5784	0.741
**PC(34:1)**	0.44071	0.39755	0.30768	0.54972	0.0683	0.2	0.43493	-0.0109	0.83226	1	0.34994	0.30323	-0.33348	-0.27164	0.32547	0.33259	0.17998	-0.46963	-0.23204	-0.21335	0.04516	-0.08254	0.20801
**PC(34:1)**	0.0148	0.0296	0.0981	0.0017	0.7199	0.2893	0.0163	0.9544	<.0001		0.058	0.1033	0.0717	0.1465	0.0792	0.0725	0.3413	0.0088	0.2173	0.2576	0.8127	0.6646	0.27
**PC(36:1)**	0.30189	0.20667	-0.32547	0.32414	-0.15328	-0.36596	0.15595	0.04338	0.15417	0.34994	1	0.47809	-0.38687	-0.37842	0.17197	0.34905	0.23515	-0.45628	-0.14438	-0.06162	0.09499	-0.07942	0.30812
**PC(36:1)**	0.1049	0.2732	0.0792	0.0806	0.4187	0.0467	0.4105	0.8199	0.416	0.058		0.0075	0.0347	0.0392	0.3635	0.0587	0.211	0.0113	0.4465	0.7463	0.6175	0.6765	0.0976
**SM(34:1)**	0.25873	0.06652	-0.12925	0.34994	0.2614	-0.08031	0.15684	-0.22981	0.08031	0.30323	0.47809	1	-0.33793	-0.35662	0.11368	0.33037	0.24983	-0.30679	-0.10612	-0.2	-0.08031	0.04917	0.31702
**SM(34:1)**	0.1674	0.7269	0.496	0.058	0.1629	0.6731	0.4078	0.2218	0.6731	0.1033	0.0075		0.0678	0.0531	0.5497	0.0746	0.183	0.0992	0.5768	0.2893	0.6731	0.7964	0.0878
**PI(34:1)**	-0.20178	-0.14527	-0.11502	-0.13949	-0.19288	0.06296	-0.05717	0.43715	-0.11635	-0.33348	-0.38687	-0.33793	1	0.94705	-0.5782	-0.75172	-0.48031	0.18042	0.08343	0.49855	-0.21646	0.11146	-0.75617
**PI(34:1)**	0.2849	0.4437	0.545	0.4622	0.3072	0.741	0.7641	0.0157	0.5403	0.0717	0.0347	0.0678		<.0001	0.0008	<.0001	0.0072	0.34	0.6612	0.005	0.2506	0.5576	<.0001
**PI(36:1)**	-0.23293	-0.09588	-0.06073	-0.17019	-0.18087	0.14483	-0.08165	0.40556	-0.04605	-0.27164	-0.37842	-0.35662	0.94705	1	-0.58532	-0.75083	-0.51769	0.20534	0.1426	0.48076	-0.29477	0.10167	-0.78287
**PI(36:1)**	0.2155	0.6142	0.7499	0.3686	0.3388	0.4451	0.668	0.0262	0.8091	0.1465	0.0392	0.0531	<.0001		0.0007	<.0001	0.0034	0.2764	0.4522	0.0072	0.1138	0.5929	<.0001
**$C_{20:4}$**	0.56618	0.65072	0.28632	0.30145	0.19644	0.17241	0.62759	0.05762	0.07898	0.32547	0.17197	0.11368	-0.5782	-0.58532	1	0.77175	0.31791	-0.30723	-0.4594	-0.6307	-0.26318	-0.50078	0.41535
**$C_{20:4}$**	0.0011	<.0001	0.1251	0.1055	0.2981	0.3623	0.0002	0.7623	0.6783	0.0792	0.3635	0.5497	0.0008	0.0007		<.0001	0.0869	0.0986	0.0107	0.0002	0.16	0.0048	0.0225
**$C_{22:4}$**	0.28676	0.32948	0.10523	0.27253	0.08209	-0.05984	0.23159	-0.32369	0.16307	0.33259	0.34905	0.33037	-0.75172	-0.75083	0.77175	1	0.48743	-0.29655	-0.28009	-0.51101	-0.00957	-0.39088	0.71435
**$C_{22:4}$**	0.1244	0.0754	0.58	0.1451	0.6663	0.7534	0.2182	0.081	0.3892	0.0725	0.0587	0.0746	<.0001	<.0001	<.0001		0.0063	0.1115	0.1338	0.0039	0.96	0.0327	<.0001
**$C_{22:6}$**	0.12303	-0.05717	0.09811	0.05451	0.02469	0.01758	0.07987	-0.19867	0.13326	0.17998	0.23515	0.24983	-0.48031	-0.51769	0.31791	0.48743	1	-0.28899	-0.65384	-0.51813	-0.17775	-0.11902	0.53281
**$C_{22:6}$**	0.5172	0.7641	0.606	0.7748	0.8969	0.9266	0.6748	0.2926	0.4827	0.3413	0.211	0.183	0.0072	0.0034	0.0869	0.0063		0.1214	<.0001	0.0034	0.3474	0.531	0.0024
**PC(38:4)**	-0.32191	-0.29344	0.09143	-0.53904	0.41135	0.05317	-0.34727	-0.10567	-0.33749	-0.46963	-0.45628	-0.30679	0.18042	0.20534	-0.30723	-0.29655	-0.28899	1	0.4812	0.20756	0.00912	0.18977	-0.22981
**PC(38:4)**	0.0828	0.1155	0.6308	0.0021	0.0239	0.7802	0.0601	0.5784	0.0682	0.0088	0.0113	0.0992	0.34	0.2764	0.0986	0.1115	0.1214		0.0071	0.2711	0.9618	0.3152	0.2218
**PI(38:4)**	-0.53993	-0.43582	-0.26674	-0.20712	-0.05362	-0.26808	-0.60133	-0.39844	-0.13815	-0.23204	-0.14438	-0.10612	0.08343	0.1426	-0.4594	-0.28009	-0.65384	0.4812	1	0.43893	0.3842	0.33526	-0.0812
**PI(38:4)**	0.0021	0.0161	0.1542	0.2721	0.7784	0.1521	0.0004	0.0292	0.4666	0.2173	0.4465	0.5768	0.6612	0.4522	0.0107	0.1338	<.0001	0.0071		0.0152	0.0361	0.0701	0.6697
**PE(36:4)**	-0.42158	-0.42514	-0.24672	-0.06118	-0.29922	-0.13415	-0.36062	-0.04027	-0.02514	-0.21335	-0.06162	-0.2	0.49855	0.48076	-0.6307	-0.51101	-0.51813	0.20756	0.43893	1	0.40111	0.52036	-0.25962
**PE(36:4)**	0.0203	0.0192	0.1887	0.7481	0.1082	0.4797	0.0503	0.8327	0.8951	0.2576	0.7463	0.2893	0.005	0.0072	0.0002	0.0039	0.0034	0.2711	0.0152		0.028	0.0032	0.1659
**PE(38:4)**	-0.13993	-0.26763	-0.18799	0.16263	-0.18265	-0.24182	-0.36997	-0.13771	0.02558	0.04516	0.09499	-0.08031	-0.21646	-0.29477	-0.26318	-0.00957	-0.17775	0.00912	0.3842	0.40111	1	0.37219	0.13415
**PE(38:4)**	0.4608	0.1528	0.3198	0.3905	0.334	0.1979	0.0442	0.468	0.8932	0.8127	0.6175	0.6731	0.2506	0.1138	0.16	0.96	0.3474	0.9618	0.0361	0.028		0.0428	0.4797
**PE(P-38:5)**	-0.42558	-0.59644	-0.18843	0.0416	-0.16796	-0.17108	-0.43715	-0.28988	-0.10567	-0.08254	-0.07942	0.04917	0.11146	0.10167	-0.50078	-0.39088	-0.11902	0.18977	0.33526	0.52036	0.37219	1	0.14127
**PE(P-38:5)**	0.019	0.0005	0.3187	0.8272	0.375	0.366	0.0157	0.1202	0.5784	0.6646	0.6765	0.7964	0.5576	0.5929	0.0048	0.0327	0.531	0.3152	0.0701	0.0032	0.0428		0.4565
**PE(O-38:5)**	-0.1475	-0.22714	0.03226	0.20356	-0.01891	-0.2485	-0.10834	-0.73882	0.06296	0.20801	0.30812	0.31702	-0.75617	-0.78287	0.41535	0.71435	0.53281	-0.22981	-0.0812	-0.25962	0.13415	0.14127	1
**PE(O-38:5)**	0.4367	0.2274	0.8656	0.2806	0.921	0.1855	0.5688	<.0001	0.741	0.27	0.0976	0.0878	<.0001	<.0001	0.0225	<.0001	0.0024	0.2218	0.6697	0.1659	0.4797	0.4565	

Supplementary Table S11 Correlation of characteristic lipids in MCF-361 cell line (correlation coefficient and *p* values were listed between every two lipid

	$C_{16:0}$	$C_{18:0}$	PC(28:0)	PC(30:0)	PC(32:0)	SM(34:0)	$C_{16:1}$	$C_{18:1}$	PC(32:1)	PC(34:1)	PC(36:1)	SM(34:1)	PI(34:1)	PI(36:1)	$C_{20:4}$	$C_{22:4}$	$C_{22:6}$	PC(38:4)	PI(38:4)	PE(36:4)	PE(38:4)	PE(P-38:5)	PE(O-38:5)
**$C_{16:0}$**	1	0.89364	-0.24274	-0.0069	0.23696	-0.10457	0.10325	-0.0445	0.0514	0.04984	0.28079	0.04138	-0.48776	-0.70271	0.83222	0.5968	0.83445	0.01046	-0.21184	0.43124	0.50734	-0.11615	-0.24299
**$C_{16:0}$**		<.0001	0.1962	0.9711	0.2074	0.5824	0.5872	0.8154	0.7874	0.7937	0.1328	0.8281	0.0063	<.0001	<.0001	0.0005	<.0001	0.9563	0.2611	0.0173	0.0042	0.541	0.1957
**$C_{18:0}$**	0.89364	1	-0.06653	0.02781	0.2601	-0.05607	-0.15977	-0.28838	0.07409	-0.11169	0.0623	0.19802	-0.34579	-0.81353	0.77348	0.42857	0.77081	-0.02514	0.03738	0.56475	0.51135	-0.26791	-0.40009
**$C_{18:0}$**	<.0001		0.7269	0.884	0.1651	0.7685	0.399	0.1222	0.6972	0.5568	0.7436	0.2942	0.0612	<.0001	<.0001	0.0181	<.0001	0.8951	0.8445	0.0011	0.0039	0.1523	0.0285
**PC(28:0)**	-0.24274	-0.06653	1	0.10478	-0.19511	0.37575	-0.00111	0.13683	0.2396	-0.19377	-0.23915	0.21602	-0.10969	0.06297	0.03716	-0.05718	-0.19112	-0.28587	-0.0376	-0.03004	-0.04205	0.11102	0.091
**PC(28:0)**	0.1962	0.7269		0.5816	0.3015	0.0407	0.9953	0.4709	0.2022	0.3049	0.2031	0.2516	0.5639	0.741	0.8454	0.7641	0.3117	0.1257	0.8436	0.8748	0.8254	0.5592	0.6325
**PC(30:0)**	-0.0069	0.02781	0.10478	1	0.40378	0.37442	-0.20714	-0.14796	-0.19867	-0.21513	-0.08832	0.23693	0.08299	-0.01802	-0.07721	-0.08433	0.02292	0.38643	0.10568	0.02158	-0.16487	0.0465	-0.28546
**PC(30:0)**	0.9711	0.884	0.5816		0.0269	0.0415	0.2721	0.4352	0.2926	0.2536	0.6426	0.2074	0.6628	0.9247	0.6851	0.6577	0.9043	0.0349	0.5783	0.9099	0.384	0.8072	0.1262
**PC(32:0)**	0.23696	0.2601	-0.19511	0.40378	1	-0.0465	-0.31216	-0.37268	-0.66274	-0.36107	0.03137	-0.02425	-0.09501	-0.35399	0.06875	0.03983	0.20536	0.6921	0.1533	0.20714	-0.09145	-0.00467	-0.12615
**PC(32:0)**	0.2074	0.1651	0.3015	0.0269		0.8072	0.0931	0.0425	<.0001	0.05	0.8693	0.8988	0.6175	0.055	0.7181	0.8345	0.2763	<.0001	0.4186	0.2721	0.6308	0.9805	0.5065
**SM(34:0)**	-0.10457	-0.05607	0.37575	0.37442	-0.0465	1	0.07965	0.17266	0.0941	-0.13504	-0.1386	0.32547	-0.09078	0.19846	-0.01513	0.09078	-0.12816	-0.29566	-0.05918	-0.18423	-0.08054	-0.00222	0.05785
**SM(34:0)**	0.5824	0.7685	0.0407	0.0415	0.8072		0.6757	0.3616	0.6209	0.4768	0.4651	0.0792	0.6333	0.2931	0.9368	0.6333	0.4997	0.1127	0.7561	0.3298	0.6722	0.9907	0.7614
**$C_{16:1}$**	0.10325	-0.15977	-0.00111	-0.20714	-0.31216	0.07965	1	0.90877	0.08121	0.25008	0.2127	0.05206	-0.4753	0.40053	0.28393	0.45038	0.08367	-0.29347	-0.90565	-0.38896	0.09657	0.39119	0.65732
**$C_{16:1}$**	0.5872	0.399	0.9953	0.2721	0.0931	0.6757		<.0001	0.6697	0.1826	0.2591	0.7847	0.0079	0.0283	0.1284	0.0125	0.6603	0.1155	<.0001	0.0336	0.6117	0.0325	<.0001
**$C_{18:1}$**	-0.0445	-0.28838	0.13683	-0.14796	-0.37268	0.17266	0.90877	1	0.10479	0.24608	0.10146	-0.03515	-0.41834	0.50512	0.18781	0.31331	-0.0494	-0.32417	-0.88696	-0.46239	0.07833	0.46907	0.66177
**$C_{18:1}$**	0.8154	0.1222	0.4709	0.4352	0.0425	0.3616	<.0001		0.5816	0.1899	0.5937	0.8537	0.0214	0.0044	0.3203	0.0918	0.7955	0.0805	<.0001	0.0101	0.6808	0.0089	<.0001
**PC(32:1)**	0.0514	0.07409	0.2396	-0.19867	-0.66274	0.0941	0.08121	0.10479	1	0.40512	-0.17108	0.13504	0.08344	0.04161	0.08789	0.01936	0.15285	-0.59199	-0.0158	-0.05896	0.07943	-0.10702	-0.11503
**PC(32:1)**	0.7874	0.6972	0.2022	0.2926	<.0001	0.6209	0.6697	0.5816		0.0264	0.366	0.4768	0.6611	0.8272	0.6442	0.9191	0.42	0.0006	0.934	0.7569	0.6765	0.5735	0.545
**PC(34:1)**	0.04984	-0.11169	-0.19377	-0.21513	-0.36107	-0.13504	0.25008	0.24608	0.40512	1	0.30456	-0.34549	0.13528	0.3244	-0.04405	0.07787	-0.11659	-0.13148	-0.13839	-0.36267	-0.14151	-0.16954	0.00311
**PC(34:1)**	0.7937	0.5568	0.3049	0.2536	0.05	0.4768	0.1826	0.1899	0.0264		0.1018	0.0615	0.476	0.0803	0.8172	0.6825	0.5395	0.4886	0.4658	0.0489	0.4557	0.3704	0.987
**PC(36:1)**	0.28079	0.0623	-0.23915	-0.08832	0.03137	-0.1386	0.2127	0.10146	-0.17108	0.30456	1	-0.09499	-0.17755	-0.093	0.26788	0.25409	0.07787	0.1297	-0.16287	0.05384	0.05117	0.04806	0.04539
**PC(36:1)**	0.1328	0.7436	0.2031	0.6426	0.8693	0.4651	0.2591	0.5937	0.366	0.1018		0.6175	0.3479	0.625	0.1524	0.1754	0.6825	0.4945	0.3898	0.7775	0.7883	0.8009	0.8118
**SM(34:1)**	0.04138	0.19802	0.21602	0.23693	-0.02425	0.32547	0.05206	-0.03515	0.13504	-0.34549	-0.09499	1	-0.30437	-0.29992	0.26299	0.16064	0.12015	-0.40601	-0.03827	0.3689	0.19001	0.19713	0.18378
**SM(34:1)**	0.8281	0.2942	0.2516	0.2074	0.8988	0.0792	0.7847	0.8537	0.4768	0.0615	0.6175		0.102	0.1073	0.1603	0.3964	0.5271	0.026	0.8409	0.0449	0.3146	0.2964	0.331
**PI(34:1)**	-0.48776	-0.34579	-0.10969	0.08299	-0.09501	-0.09078	-0.4753	-0.41834	0.08344	0.13528	-0.17755	-0.30437	1	0.42145	-0.78015	-0.68803	-0.49088	0.24141	0.63996	-0.34713	-0.59368	-0.61638	-0.55007
**PI(34:1)**	0.0063	0.0612	0.5639	0.6628	0.6175	0.6333	0.0079	0.0214	0.6611	0.476	0.3479	0.102		0.0204	<.0001	<.0001	0.0059	0.1987	0.0001	0.0602	0.0005	0.0003	0.0016
**PI(36:1)**	-0.70271	-0.81353	0.06297	-0.01802	-0.35399	0.19846	0.40053	0.50512	0.04161	0.3244	-0.093	-0.29992	0.42145	1	-0.68847	-0.27503	-0.71518	0.00734	-0.22563	-0.82733	-0.57098	0.04539	0.26702
**PI(36:1)**	<.0001	<.0001	0.741	0.9247	0.055	0.2931	0.0283	0.0044	0.8272	0.0803	0.625	0.1073	0.0204		<.0001	0.1413	<.0001	0.9693	0.2306	<.0001	0.001	0.8117	0.1537
**$C_{20:4}$**	0.83222	0.77348	0.03716	-0.07721	0.06875	-0.01513	0.28393	0.18781	0.08789	-0.04405	0.26788	0.26299	-0.78015	-0.68847	1	0.63685	0.79884	-0.24586	-0.43569	0.51847	0.65999	0.20739	0.14953
**$C_{20:4}$**	<.0001	<.0001	0.8454	0.6851	0.7181	0.9368	0.1284	0.3203	0.6442	0.8172	0.1524	0.1603	<.0001	<.0001		0.0002	<.0001	0.1903	0.0161	0.0033	<.0001	0.2715	0.4303
**$C_{22:4}$**	0.5968	0.42857	-0.05718	-0.08433	0.03983	0.09078	0.45038	0.31331	0.01936	0.07787	0.25409	0.16064	-0.68803	-0.27503	0.63685	1	0.4348	-0.1751	-0.51446	0.07388	0.31687	0.31642	0.21406
**$C_{22:4}$**	0.0005	0.0181	0.7641	0.6577	0.8345	0.6333	0.0125	0.0918	0.9191	0.6825	0.1754	0.3964	<.0001	0.1413	0.0002		0.0163	0.3547	0.0036	0.698	0.088	0.0885	0.256
**$C_{22:6}$**	0.83445	0.77081	-0.19112	0.02292	0.20536	-0.12816	0.08367	-0.0494	0.15285	-0.11659	0.07787	0.12015	-0.49088	-0.71518	0.79884	0.4348	1	-0.04784	-0.23854	0.54072	0.49266	-0.00935	-0.07477
**$C_{22:6}$**	<.0001	<.0001	0.3117	0.9043	0.2763	0.4997	0.6603	0.7955	0.42	0.5395	0.6825	0.5271	0.0059	<.0001	<.0001	0.0163		0.8018	0.2043	0.002	0.0057	0.9609	0.6946
**PC(38:4)**	0.01046	-0.02514	-0.28587	0.38643	0.6921	-0.29566	-0.29347	-0.32417	-0.59199	-0.13148	0.1297	-0.40601	0.24141	0.00734	-0.24586	-0.1751	-0.04784	1	0.22583	0.03894	-0.25164	-0.01847	-0.31305
**PC(38:4)**	0.9563	0.8951	0.1257	0.0349	<.0001	0.1127	0.1155	0.0805	0.0006	0.4886	0.4945	0.026	0.1987	0.9693	0.1903	0.3547	0.8018		0.2302	0.8381	0.1798	0.9228	0.0921
**PI(38:4)**	-0.21184	0.03738	-0.0376	0.10568	0.1533	-0.05918	-0.90565	-0.88696	-0.0158	-0.13839	-0.16287	-0.03827	0.63996	-0.22563	-0.43569	-0.51446	-0.23854	0.22583	1	0.25456	-0.21006	-0.49266	-0.6235
**PI(38:4)**	0.2611	0.8445	0.8436	0.5783	0.4186	0.7561	<.0001	<.0001	0.934	0.4658	0.3898	0.8409	0.0001	0.2306	0.0161	0.0036	0.2043	0.2302		0.1746	0.2652	0.0057	0.0002
**PE(36:4)**	0.43124	0.56475	-0.03004	0.02158	0.20714	-0.18423	-0.38896	-0.46239	-0.05896	-0.36267	0.05384	0.3689	-0.34713	-0.82733	0.51847	0.07388	0.54072	0.03894	0.25456	1	0.60392	0.16422	-0.16511
**PE(36:4)**	0.0173	0.0011	0.8748	0.9099	0.2721	0.3298	0.0336	0.0101	0.7569	0.0489	0.7775	0.0449	0.0602	<.0001	0.0033	0.698	0.002	0.8381	0.1746		0.0004	0.3859	0.3833
**PE(38:4)**	0.50734	0.51135	-0.04205	-0.16487	-0.09145	-0.08054	0.09657	0.07833	0.07943	-0.14151	0.05117	0.19001	-0.59368	-0.57098	0.65999	0.31687	0.49266	-0.25164	-0.21006	0.60392	1	0.27058	0.07165
**PE(38:4)**	0.0042	0.0039	0.8254	0.384	0.6308	0.6722	0.6117	0.6808	0.6765	0.4557	0.7883	0.3146	0.0005	0.001	<.0001	0.088	0.0057	0.1798	0.2652	0.0004		0.1481	0.7067
**PE(P-38:5)**	-0.11615	-0.26791	0.11102	0.0465	-0.00467	-0.00222	0.39119	0.46907	-0.10702	-0.16954	0.04806	0.19713	-0.61638	0.04539	0.20739	0.31642	-0.00935	-0.01847	-0.49266	0.16422	0.27058	1	0.59012
**PE(P-38:5)**	0.541	0.1523	0.5592	0.8072	0.9805	0.9907	0.0325	0.0089	0.5735	0.3704	0.8009	0.2964	0.0003	0.8117	0.2715	0.0885	0.9609	0.9228	0.0057	0.3859	0.1481		0.0006
**PE(O-38:5)**	-0.24299	-0.40009	0.091	-0.28546	-0.12615	0.05785	0.65732	0.66177	-0.11503	0.00311	0.04539	0.18378	-0.55007	0.26702	0.14953	0.21406	-0.07477	-0.31305	-0.6235	-0.16511	0.07165	0.59012	1
**PE(O-38:5)**	0.1957	0.0285	0.6325	0.1262	0.5065	0.7614	<.0001	<.0001	0.545	0.987	0.8118	0.331	0.0016	0.1537	0.4303	0.256	0.6946	0.0921	0.0002	0.3833	0.7067	0.0006	

Supplementary Table S12. The p values obtained using one way ANOVA analysis beween two cell lines

	CKα	SMS1	FASN1	SCD1
MCF-10A vs. BT-20	0.046778279	0.024211112	3.84E-07	0.573073174
MCF-10A vs. MCF-7	0.011855225	0.034811495	5.64E-06	1.44E-07
MCF-10A vs. SK-BR-3	0.002177613	0.049952255	1.51E-06	0.523620597
MCF-10A vs. MDA-MB-231	6.99E-05	0.957093003	0.924542761	0.421534864
MCF-10A vs. MDA-MB-157	0.579126654	0.552965135	0.552453881	0.458981122
MCF-10A vs. MDA-MB-361	0.011136204	0.001001223	1.81E-07	0.223274387
BT-20 vs. MCF-7	0.381692973	0.852735214	0.086664452	7.03E-08
BT-20 vs. SK-BR-3	0.100801858	0.709036326	0.345168001	0.93963507
BT-20 vs. MDA-MB-231	0.003043464	0.036913228	3.37E-07	0.805456147
BT-20 vs. MDA-MB-157	0.022873731	0.015711696	1.72E-07	0.856282018
BT-20 vs. MDA-MB-361	2.30E-04	0.128901951	0.579244204	0.49700635
MCF-7 vs. SK-BR-3	0.407734299	0.850689926	0.401285229	6.41E-08
MCF-7 vs. MDA-MB-231	0.018221477	0.048625405	4.85E-06	5.20E-08
MCF-7 vs. MDA-MB-157	0.003837894	0.040576546	2.22E-06	5.63E-08
MCF-7 vs. MDA-MB-361	4.50E-05	0.045984815	0.030267469	3.08E-08
SK-BR-3 vs. MDA-MB-231	0.090413893	0.035293157	1.32E-06	0.864429003
SK-BR-3 vs. MDA-MB-157	7.16E-04	0.011464937	6.35E-07	0.916010641
SK-BR-3 vs. MDA-MB-361	1.07E-05	0.065942295	0.144732594	0.545062509
MDA-MB-231 vs. MDA-MB-157	2.60E-05	0.588877281	0.61643092	0.947900384
MDA-MB-231 vs. MDA-MB-361	6.85E-07	0.001114195	1.60E-07	0.662179865
MDA-MB-157 vs. MDA-MB-361	0.033665904	0.003318149	8.42E-08	0.616044249

P values highlighted in red represent no statistical differences.
